# Effectiveness of Targeted Interventions on Treatment of Infants With Bronchiolitis: A Randomized Clinical Trial

**DOI:** 10.1001/jamapediatrics.2021.0295

**Published:** 2021-04-12

**Authors:** Libby Haskell, Emma J. Tavender, Catherine L. Wilson, Sharon O’Brien, Franz E. Babl, Meredith L. Borland, Elizabeth Cotterell, Rachel Schembri, Francesca Orsini, Nicolette Sheridan, David W. Johnson, Ed Oakley, Stuart R. Dalziel

**Affiliations:** 1 Children’s Emergency Department, Starship Children’s Hospital, Auckland, New Zealand; 2 Emergency Research, Murdoch Children’s Research Institute, Victoria, Australia; 3 Department of Pediatrics, University of Melbourne, Victoria, Australia; 4 Emergency Department, Perth Children’s Hospital, Western Australia, Australia; 5 Curtin University, Bentley, Western Australia, Australia; 6 Pediatric Emergency Medicine, University of Melbourne, Victoria, Australia; 7 The Royal Children’s Hospital Melbourne, Victoria, Australia; 8 Faculty of Health and Medical Sciences (Pediatrics and Emergency Medicine), The University of Western Australia, Western Australia; 9 Armidale Rural Referral Hospital, New South Wales, Australia; 10 School of Rural Medicine, University of New England, New South Wales, Australia; 11 Clinical Epidemiology and Biostatistics, Melbourne Children's Trials Center, Murdoch Children’s Research Institute, Victoria, Australia; 12 Center for Nursing and Health Research, Massey University, Auckland, Auckland, New Zealand; 13 Department of Pediatrics, Cumming School of Medicine, University of Calgary, Calgary, Alberta, Canada; 14 University of Melbourne, Victoria, Australia; 15 Departments of Surgery and Pediatrics, Child and Youth Health, The University of Auckland, Auckland, New Zealand

## Abstract

**Importance:**

In developed countries, bronchiolitis is the most common reason for infants to be admitted to the hospital, and all international bronchiolitis guidelines recommend supportive care; however, significant variation in practice continues with infants receiving non–evidence-based therapies. Deimplementation research aims to reduce the use of low-value care, and advancing science in this area is critical to delivering evidence-based care.

**Objective:**

To determine the effectiveness of targeted interventions vs passive dissemination of an evidence-based bronchiolitis guideline in improving treatment of infants with bronchiolitis.

**Design, Setting, and Participants:**

This international, multicenter cluster randomized clinical trial included 26 hospitals (clusters) in Australia and New Zealand providing tertiary or secondary pediatric care (13 randomized to intervention, 13 to control) during the 2017 bronchiolitis season. Data were collected on 8003 infants for the 3 bronchiolitis seasons (2014-2016) before the implementation period and 3727 infants for the implementation period (2017 bronchiolitis season, May 1-November 30). Data were analyzed from November 16, 2018, to December 9, 2020.

**Interventions:**

Interventions were developed using theories of behavior change to target key factors that influence bronchiolitis management. These interventions included site-based clinical leads, stakeholder meetings, a train-the-trainer workshop, targeted educational delivery, other educational and promotional materials, and audit and feedback.

**Main Outcomes and Measures:**

The primary outcome was compliance during the first 24 hours of care with no use of chest radiography, albuterol, glucocorticoids, antibiotics, and epinephrine, measured retrospectively from medical records of randomly selected infants with bronchiolitis who presented to the hospital. There were no patient-level exclusions.

**Results:**

A total of 26 hospitals were randomized without dropouts. Analysis was by intention to treat. Baseline data collected on 8003 infants for 3 bronchiolitis seasons (2014-2016) before the implementation period were similar between intervention and control hospitals. Implementation period data were collected on 3727 infants, including 2328 boys (62%) and 1399 girls (38%), with a mean (SD) age of 6.0 (3.2) months. A total of 459 (12%) were Māori (New Zealand), and 295 (8%) were Aboriginal/Torres Strait Islander (Australia). Compliance with recommendations was 85.1% (95% CI, 82.6%-89.7%) in intervention hospitals vs 73.0% (95% CI, 65.3%-78.8%) in control hospitals (adjusted risk difference, 14.1%; 95% CI, 6.5%-21.7%; *P* < .001).

**Conclusions and Relevance:**

Targeted interventions led to improved treatment of infants with bronchiolitis. This study has important implications for bronchiolitis management and the development of effective interventions to deimplement low-value care.

**Trial Registration:**

Australian and New Zealand Clinical Trials Registry: ACTRN12616001567415.

## Key Points

**Question:**

Can the evidence-based treatment of infants with bronchiolitis be improved by using targeted interventions to deimplement low-value care?

**Findings:**

In this international cluster randomized clinical trial of 26 hospitals and 3727 infants, an absolute risk difference favoring intervention hospitals was seen in compliance with 5 evidence-based recommendations in the treatment of infants with bronchiolitis.

**Meaning:**

Use of targeted interventions improved the treatment of infants with bronchiolitis by deimplementing the use of ineffective and potentially harmful therapies and management; these results are important for bronchiolitis management, deimplementation science, and future interventions in acute care pediatrics.

## Teaser

This cluster randomized clinical trial uses data from 26 hospitals to investigate whether evidence-based treatment of infants with bronchiolitis can be improved by using targeted interventions to deimplement low-value care.

## Introduction

Bronchiolitis is the most common respiratory condition affecting infants. In developed countries, bronchiolitis is the leading cause of admission to the hospital for infants,^1^ with infants from indigenous and impoverished communities being most at risk.^2^ This increased risk is, in part, the effect of structural racism resulting in indigenous populations being more likely to live in poverty and have reduced health services access, which leads to worse health outcomes.^3^ Bronchiolitis presentations occur at secondary as well as tertiary hospitals, and management occurs in both emergency departments (EDs) and pediatric wards; hence, evidence-based treatment needs to penetrate all levels of hospital and health systems.^4,5^ Hospital admission costs alone are estimated in the US to exceed $1.7 billion per year.^1,6,7^

Management of bronchiolitis is well defined internationally.^1^ Guidelines recommend respiratory and hydration support,^8,9,10,11,12^ and they recommend against the use of chest radiography (CR), albuterol, glucocorticoids, antibiotics, and epinephrine (eTable 1 in Supplement 1). Despite evidence that these 5 therapies and management processes are ineffective and associated with harm,^11,12,13^ they continue to be widely used. In Australia and New Zealand (termed Australasia by the people of Australia and New Zealand), data from more than 3400 infant presentations to 7 hospitals demonstrated that at least 1 of these 5 interventions was used at least once in 27% to 48% of bronchiolitis admissions.^14^ These data are consistent with comparisons in North America, the United Kingdom, and Europe,^15^ highlighting the gap between the evidence-based and current clinical practices that exist internationally.

Implementation science aims to assess the effectiveness of interventions in translating research knowledge, reducing the gap between evidence-based practice and current clinical practice.^16^ Dissemination of a clinical practice guideline is seldom sufficient to change practice,^17^ with more active strategies being required to effect change. Strategies are more likely to be effective if underpinned by theories of behavior change and address both the barriers to and enhancers of recommended practice.^18,19^ The Theoretical Domains Framework (TDF) incorporates a range of behavior change theories for use in implementation research.^20^ The validated TDF^21^ has demonstrated strong explanatory and predictive powers across health care, including acute care settings, and is particularly useful when selecting interventions to improve practice.^22,23^ Benefits of the TDF are that each domain has behavior change techniques linked to it, thereby optimizing use of techniques most likely to tackle identified issues,^24^ with guidance available to assist in its use.^25^ It was for all of these reasons that the TDF was used in our study. Less attention has focused on the challenge of reducing low-value health care and developing a theory and evidence to support deimplementation, which is of considerable importance for health care systems.^26,27^

To our knowledge, no randomized clinical trials (RCTs) have been reported to determine the effectiveness of targeted interventions on bronchiolitis management.^28^ Similarly, RCTs to deimplement unnecessary care in the acute care arena are rare. We undertook a cluster RCT to determine the effectiveness of targeted interventions vs passive dissemination of a clinical guideline to improve the evidence-based clinical care of infants presenting with bronchiolitis to the hospital. Minimizing harm caused by unnecessary interventions in the management of infants with bronchiolitis is an important patient- and family-centered outcome, and it is key to health care systems delivering evidence-based, cost-effective clinical management.

## Methods

### Design

A detailed trial protocol has been published previously and is available in Supplement 2.^29^ This study was a multicenter cluster RCT. Participating hospitals (clusters) were randomized between December 21, 2016, and February 3, 2017. The study was approved by the Royal Children’s Hospital Human Research Ethics Committee, Australia (HREC/16/RCHM/84), and the Northern A Health and Disability Ethics Committee, New Zealand (16/NTA/146). The ethics committee waived the requirement for informed patient consent because unidentified data from the medical records were being collected. This study followed the Consolidated Standards of Reporting Trials (CONSORT) reporting guideline.

### Setting

This study included 26 hospitals (clusters) in Australia and New Zealand. Hospitals were eligible if they (1) had more than 135 bronchiolitis presentations per year, (2) were willing to be randomized to control or intervention, (3) had signed agreements from the ED and pediatric inpatient clinical directors, and (4) could retrospectively collect required data. Study design and recruitment are outlined in the Figure.

**Figure.**
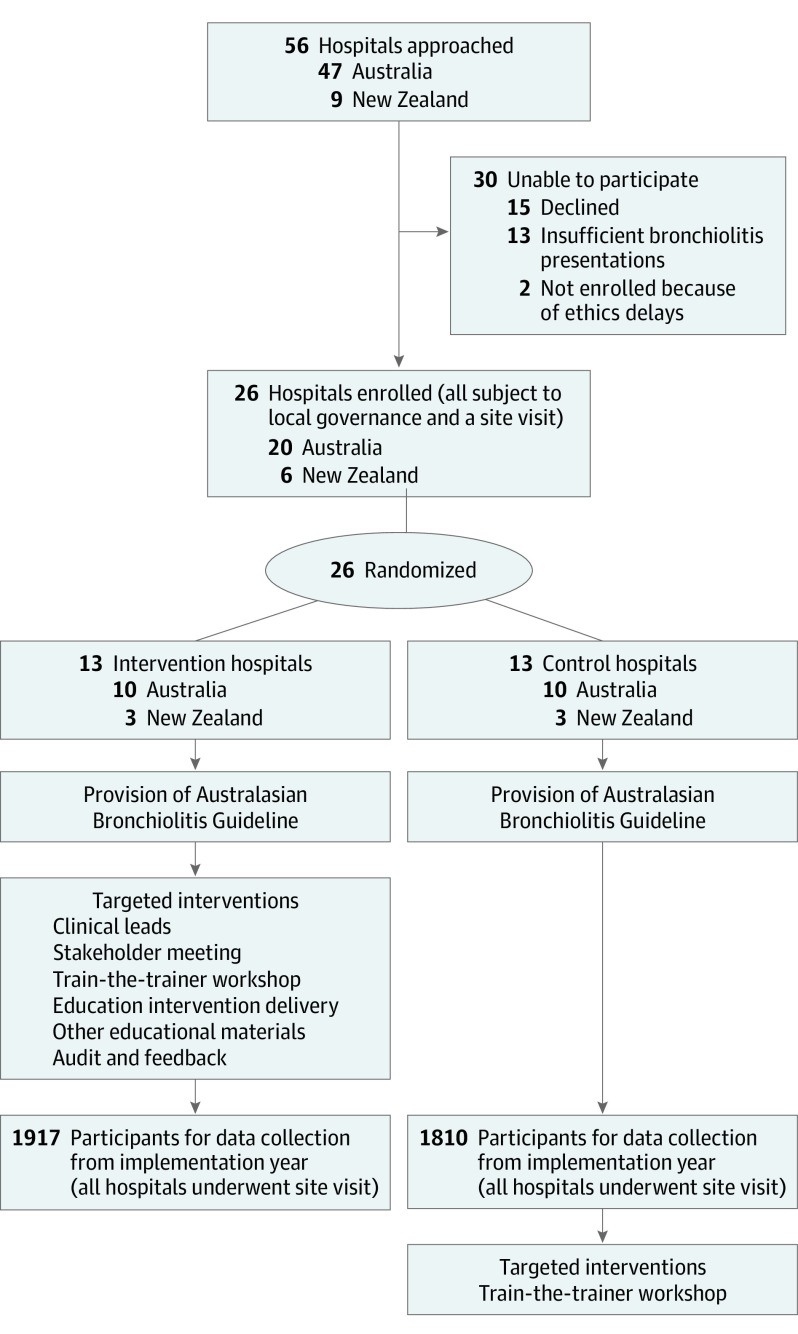
Consolidated Standards of Reporting Trials (CONSORT) Flow Diagram and Study Design

### Randomization, Masking, and Participants

Following hospital consent, an independent statistician randomized hospitals using Stata software, version 14.2 (StataCorp LLC), stratifying by country and level of pediatric care (tertiary hospitals with dedicated intensive care units [ICUs] or secondary hospitals; randomization block size 2). Owing to the type of intervention, blinding was not possible.

Infants were eligible for inclusion if they were younger than 1 year at presentation and had both an ED and discharge diagnosis of bronchiolitis. Infant age younger than 1 year is consistent with bronchiolitis recommendations in Australia and New Zealand.^11^ There were no other patient-level exclusion criteria.

### Interventions

Intervention hospitals received interventions targeting nursing and medical clinicians who managed infants with bronchiolitis in the ED and pediatric inpatient wards. Specific targeted interventions were developed using the TDF framework following a qualitative study^30^ identifying local barriers and enablers to evidence-based bronchiolitis care. Table 1 details intervention components, eFigure 1 in Supplement 1 shows the stepped development approach, and the eAppendix in Supplement 1 details how interventions were rolled out according to the Template for Intervention Description and Replication checklist.^31^ The implementation period was the 2017 bronchiolitis season, May 1 to November 30, 2017.

**Table 1.** Bronchiolitis Intervention Components

Intervention	Description
Clinical leads	Four clinical leads, including 1 nursing and 1 medical lead in each of the emergency department and inpatient pediatric areas for duration of study.
	Key tasks included attending train-the-trainer 1-d workshop, leading delivery of educational intervention and other educational materials to all staff, overseeing completion of monthly audit and delivery of feedback, and coordinating study requirements.
Stakeholder meeting	Study team met with clinical leads to present Australasian Bronchiolitis Guideline, discuss international and local variation in bronchiolitis management, review local audit results, and discuss any anticipated local barriers, with the aim to gain site buy-in.
Train-the-trainer workshop	One-day workshop for clinical leads to discuss Australasian Bronchiolitis Guideline and evidence underpinning recommendations, implementation, qualitative study identifying barriers and facilitators to bronchiolitis management, and development process of interventions. Demonstrated to clinical leads how to deliver educational intervention to their staff, outlined study data requirements and timeline, and facilitated planning time for clinical leads.
Educational intervention delivery	PowerPoint presentation designed with scripted messages addressing key findings from qualitative study using behavior change techniques most likely to effect change.
	Education delivery overseen by clinical leads to nursing and medical staff using PowerPoint presentation.
	Aimed to educate 80% of staff within first month and ongoing education throughout duration of study ensuring all staff educated.
Use of other educational materials	Clinician training video, evidence fact sheets, promotional materials, and parent/caregiver information, which were delivered locally by clinical leads.
Audit and feedback	Monthly audits of the first 20 bronchiolitis presentations, with report produced showing individual hospital results compared with top-performing site. Report disseminated by clinical leads to their staff in verbal and written format; action planning with target setting encouraged.

Control hospitals received electronic and printed copies of the Australasian Bronchiolitis Guideline,^11^ released December 2016; this was the first bronchiolitis guideline for use across Australia and New Zealand. Hospitals could undertake their usual dissemination activities. Information was not collected on how the guideline was disseminated owing to the possibility of the Hawthorne effect resulting from such a request.

### Data Collection

Data collection occurred for the implementation period and the 3 preceding years, ensuring that the baseline data and effect of temporal trends could be assessed. All infants younger than 1 year presenting to each hospital with *International Classification of Diseases, Ninth Revision (ICD-9)* and *International Statistical Classification of Diseases and Related Health Problems, Tenth Revision (ICD-10)* codes related to bronchiolitis were retrospectively identified (eTable 2 in Supplement 1). Research Electronic Data Capture (REDCap), version 8.5.1 (Vanderbilt University) was used to generate randomly selected presentations from identified lists for data extraction (n = 150 for implementation period; n = 100 for each of the previous 3 years). Auditors entered deidentified data extracted from medical records into the REDCap database, checking for eligibility. Reliability and accuracy was audited during site visits. Data included demographic details; ED and hospital or ICU length of stay; disposition; CR, albuterol, antibiotics (for respiratory cause), glucocorticoid, or any epinephrine administration during hospitalization; and death. Clinical leads at intervention hospitals maintained logs throughout the implementation period recording intervention use.

### Outcome Measures

Analysis was by intention to treat. The primary outcome was the proportion of infants who complied with all 5 Australasian Bronchiolitis Guideline^11^ recommendations known to have no benefit (CR, albuterol, glucocorticoids, antibiotics, and epinephrine) in the acute care period (first 24 hours of hospitalization). The acute care period was chosen as the primary outcome time frame because this management establishes the trajectory for further management in short-term admissions, such as bronchiolitis.

Secondary outcomes included (1) compliance with guideline recommendations (in ED, as inpatient, and during total hospitalization); (2) compliance with guideline recommendations (acute care period) for CR, albuterol, glucocorticoids, antibiotics, and epinephrine; (3) number of albuterol doses during the acute care period, during total hospitalization, while treated in the ED, and during inpatient treatment; (4) ICU admission; (5) length of hospital stay; and (6) death.

### Statistical Analysis

The primary outcome was analyzed at the individual patient level, adjusting for stratification factors at randomization (model A) and then adjusting for risk factors associated with bronchiolitis admission (model B). A priori subgroup analyses were planned if there was evidence of interaction between treatment group and subgroups: (1) infants with comorbidities vs without and (2) infants referred from another hospital or represented vs not. A priori sensitivity analyses were planned (1) at the cluster/hospital level, (2) excluding 2 hospitals without data on infants with ED length of stay less than 3 hours, and (3) using multiple imputation if primary outcome data were missing for more than 10% of the infants randomized for data extraction. Post hoc analyses were adjusted for preintervention compliance with all 5 bronchiolitis guideline recommendations (model A) to test any possible temporal trend in compliance in years preceding implementation affecting results and then adjusted for severity of illness (Australasian Triage Scale 1-2, immediately/imminently life-threatening vs 3-5, potentially life-threatening/potentially serious/less urgent).

Generalized linear models (GLMs) used the binomial family and an identity link function and calculated a cluster-robust SE to account for study site. Based on the GLM, risk differences (RDs) between guideline compliance proportions in the 2 groups and 95% CIs were computed. When GLM models did not converge, logistic regression with a cluster-robust standard error (hospital level) was used to calculate odds ratios.

Secondary outcomes were analyzed using GLM (model A). For outcomes not normally distributed, the GLM approach used the Poisson family and a log-link function and a cluster-robust SE (hospital level) to compute incidence rate ratios (IRRs) and 95% CIs.

Sample size calculation was based on the hypothesis that targeted interventions were superior to passive dissemination, resulting in an absolute increase in compliance with guideline recommendations of at least 15% in the intervention group compared with controls. This difference was clinically relevant. Previous data assumed baseline compliance of 52% to 73%.^14^ Using a conservative estimate of 50% compliance, 1620 infants per group in the implementation year were required to provide 82% power (α = .05), allowing for an average intraclass correlation coefficient (ICC) of 0.055^14^ (calculated from local data) and an average cluster size of 135 (n = 24 clusters; calculated in Stata 14.2). Allowing for possible attrition and resultant loss of power, 26 hospitals were recruited, each to report outcomes of 150 infants in the implementation year.

A statistical analysis plan (Supplement 3) was finalized before database lock. We present summary statistics as absolute and relative frequencies for categorical data and mean (SD) or median (interquartile range [IQR]) for continuous data. Differences between categorical variables are presented as RDs or odds ratios (95% CI). Differences between continuous data are presented as differences between mean (95% CI) or IRR (95% CI). All *P* values were 2-sided. Data were analyzed from November 16, 2018, to December 9, 2020.

## Results

Fifty-six hospitals were initially approached: 30 were unable to participate because they declined participation, had inadequate annual bronchiolitis presentations, or were unable to obtain timely governance or ethics approval. Twenty-six hospitals (Australian = 20; New Zealand = 6) were randomized (intervention group = 13; control group = 13), including 7 Australian tertiary pediatric hospitals (Figure). No hospitals withdrew after randomization.

Infants from intervention and control hospitals for the implementation year were well balanced in terms of baseline characteristics (Table 2), with compliance ranging from 64% to 73% and 60% to 66%, respectively, for the 3 years before the implementation year (n = 8003 infants) (eFigures 2 and 3 in Supplement 1). Overall, the 3727 implementation period participants had a mean (SD) age of 6.0 (3.2) months, with 2328 boys (62%) and 1399 girls (38%). A total of 459 (12%) identified as Māori (New Zealand) and 295 (8%) identified as Aboriginal/Torres Strait Islander (Australia); 505 (14%) had a history of prematurity (gestation <37 weeks), and 172 (5%) had comorbidities. Preadmission, at least 1 of the 5 key therapies and management processes known to have no benefit, was used in 653 infants (18%).

**Table 2.** Baseline Characteristics

Characteristic	No. (%)^a^	
	Intervention	Control
**Pediatric care**		
Tertiary	4/13 (31)	3/13 (23)
Secondary	9/13 (69)	10/13 (77)
Annual ED presentations per site in 2017, median (IQR)	61 898 (53 000-81 635)	69 391 (53 880-85 413)
Proportion of ED pediatric presentations per site, median % (IQR)	25 (20-31)	21 (20-24)
Staffing: full-time equivalent per site in January 2017, median (IQR)		
Medical ED	48 (31-61)	66 (31-77)
Nursing ED	84 (72-105)	116 (75-132)
Medical inpatient pediatrics	17 (13-30)	17 (11-20)
Nursing inpatient pediatrics	30 (22-39)	26 (21-36)
**Compliance with Australasian Bronchiolitis Guideline (preintervention)**		
During 2014		
No.	790/1238	813/1351
Mean (SD), %	64 (15)	60 (17)
During 2015		
No.	952/1378	846/1355
Mean (SD), %	69 (8)	62 (16)
During 2016		
No.	989/1350	874/1331
Mean (SD), %	73 (8)	66 (14)
**Characteristics of infants during implementation period (2017 bronchiolitis season)**		
No.	1917	1810
Age, mean (SD), mo	6 (3)	6 (3)
Female	733 (38)	666 (37)
Racial/ethnic group		
Aboriginal or Torres Strait Islander^b^	126 (7)	169 (9)
Māori^c^	234 (12)	225 (12)
Pacific^c^^,^^d^	41 (2)	27 (1)
Other	1519 (79)	1393 (77)
Medical history		
Premature birth^e^	224 (12)	281 (16)
Bronchiolitis	540 (28)	447 (25)
Eczema	73 (4)	85 (5)
Comorbidities^f^	82 (4)	90 (5)
Presentation time		
Weekday^g^	639 (33)	599 (33)
After hours^h^	996 (52)	953 (53)
Overnight^i^	281 (15)	258 (14)
Australasian Triage Scale		
1 (Immediately life-threatening)	12 (1)	27 (2)
2 (Imminently life-threatening)	610 (32)	635 (35)
3 (Potentially life-threatening)	1062 (55)	935 (52)
4 (Potentially serious)	218 (11)	162 (9)
5 (Less urgent)	2 (0.1)	11 (1)
Referral source to hospital		
General practitioner	494 (26)	433 (24)
After hours accident and medical/urgent care	54 (3)	34 (2)
Another hospital	55 (3)	73 (4)
**Characteristics of infants during implementation period (2017 bronchiolitis season)**		
No.	1917	1810
Prehospital interventions^j^		
Chest radiography	26 (1)	33 (2)
Albuterol	137 (7)	139 (8)
Glucocorticoids	101 (5)	126 (7)
Antibiotics (for respiratory cause)	102 (5)	94 (5)
Epinephrine	4 (0.2)	2 (0.1)

Abbreviations: ED, emergency department; IQR, interquartile range.

^a^ Values are expressed as No. (%) unless otherwise indicated.

^b^ Australia (only recorded in Australia; presented as percentage of total cohort).

^c^ New Zealand (only recorded in New Zealand; presented as percentage of total cohort).

^e^ Premature birth includes birth prior to 37 weeks’ gestation.

^d^ Pacific includes Samoan, Tongan, Niuean, Tokelauan, Fijian, I-Kiribati, Marshall Islander, Nauruan, Palauan, Soloman Islander, Tuvaluan, and ni-Vanuatu.

^f^ Comorbidities include congenital heart disease, chronic lung disease, chronic neurological disorder, or failure to thrive.

^g^ Weekday = Monday to Friday, 8:00 am to 4:00 pm.

^h^ After hours = Monday to Friday, 4:00 pm to 12:00 am, and Saturday and Sunday, 8:00 am to 12:00 am.

^i^ Overnight = 12:00 am to 8:00 am.

^j^ Prehospital care = primary and ambulance services care independent of the hospitals.

### Primary Outcome

Compliance with the Australasian Bronchiolitis Guideline recommendations during the acute care period (first 24 hours of hospitalization) with no use of CR, albuterol, glucocorticoids, antibiotics, and epinephrine occurred in 1631 infants (85.1%; 95% CI, 82.6%-89.7%) in the intervention group and 1321 infants (73.0%; 95% CI, 65.3%-78.8%) in the control group (adjusted RD for baseline stratification factors, 14.1%; 95% CI, 6.5%-21.7%, *P* < .001; ICC, 0.11; 95% CI, 0.06-0.19). Adjusting for risk factors for bronchiolitis admission, sensitivity analyses at cluster level, and excluding 2 hospitals without data on infants with an ED length of stay less than 3 hours made no statistical difference to results. Sensitivity analysis for missing data was not undertaken as the 10% threshold was not reached (Table 3).

**Table 3.** Primary Outcome and Subgroup Analysis

Variable	No. (%)		Adjusted (95% CI)		*P* value
	Intervention (n = 1917)	Control (n = 1810)	Risk difference, %	Odds ratio	
**Compliance during the first 24 h following presentation to ED with regard to chest radiography, albuterol, glucocorticoids, antibiotics, and epinephrine**					
Model A^a^	1631/1917 (85)	1321/1810 (73)	14.1 (6.5-21.7)	NA	<.001
Model A^a^^,^^b^	NA	NA	NA	2.4 (1.4-3.9)	.001
Model B^b^^,^^c^	1596/1873 (85)	1274/1744 (73)	NA	2.3 (1.4-3.8)	<.001
Post hoc analysis adjusted for preintervention periods^a^	1631/1917 (85)	1321/1810 (73)	10.8 (5.1-16.5)	NA	<.001
**Sensitivity analysis**					
Sites, No.	13	13	NA	NA	NA
Cluster-level analysis, mean (SD), %^a^	85 (9)	71 (16)	13.5 (4.5-22.5)	NA	.005
Excluding 2 hospitals unable to collect data if ED length of stay <3 h	1483 (84)	1223 (74)	12.0 (4.3-19.6)	NA	.002
**Subgroup analysis by presentation**					
Presence of comorbidities^c^^,^^d^	63/82 (77)	59/90 (66)	NA	NA	.51^e^
Absence of comorbidities^c^^,^^d^	1534/1792 (86)	1215/1654 (73)	NA	NA	
Referral from another hospital or re-presentation to hospital^c^^,^^f^	191/240 (80)	139/217 (64)	NA	NA	.91^e^
Primary presentation^c^	1405/1633 (86)	1135/1527 (74)	NA	NA	
Post hoc analysis for triage scale 1-2^c^	480/622 (77)	410/652 (62)	NA	NA	.67^e^
Post hoc analysis for triage scale 3-5^c^	1140/1282 (89)	880/1108 (79)	NA	NA	

Abbreviations: ED, emergency department; NA, not available; OR, odds ratio; RD, risk difference.

^a^ Model A, adjusted for stratification factors at randomization (country, on-site pediatric intensive care unit). The overall observed proportion obtained by dividing the number of patients for which guidelines were followed (summed across all sites) by the total number of patients in these sites. This proportion was a weighted average of the cluster proportions, with the weights provided by the sample size for each cluster. To test the null hypothesis of no difference between the groups, a *t* test was conducted on the observed cluster-level proportions.

^b^ OR provided for comparison where RD could not be obtained.

^c^ Model B, adjusted for a priori factors associated with increased risk of bronchiolitis admission (sex, gestational age <37 weeks, chronological age <10 weeks at presentation, indigenous race/ethnicity, presence of comorbidities, and referred from another hospital or re-presentation with bronchiolitis).

^d^ Comorbidities included failure to thrive and chronic neurologic/cardiac/lung disease.

^e^ *P* value for interaction term.

^f^ Re-presentation to hospital within a single bronchiolitis illness.

### Secondary Outcomes

Compliance was improved in the intervention group for patients in the ED (RD, 10.8%; 95% CI, 4.1%-17.4%; *P* = .002), as inpatients (RD, 8.5%; 95% CI, 2.7%-14.3%; *P* = .004) and during the total hospitalization (RD, 14.4%; 95% CI, 6.2%-22.6%; *P* < .001) (Table 4). Absolute improvements in compliance occurred for each of the 5 guideline recommendations, with strong evidence for improvement in intervention patients in the use of albuterol (RD, 9.4%; 95% CI, 5.6%-13.2%; *P* < .001) and CR (RD, 6.2%; 95% CI, 0.5%-11.9%; *P* = .03) (Table 4).

**Table 4.** Secondary Outcomes^a^

Outcome	No. (%)		Adjusted (95% CI)		*P* value
	Intervention (n = 1917)	Control (n = 1810)	Risk difference, %	Incidence rate ratio	
**Compliance for each patient presentation with no use of chest radiography, albuterol, glucocorticoids, antibiotics, and epinephrine**					
While in ED	1671 (87)	1427 (79)	10.8 (4.1 to 17.4)	NA	.002
While an inpatient^b^	1735 (91)	1499 (83)	8.5 (2.7 to 14.3)	NA	.004
During total hospitalization	1576 (82)	1265 (70)	14.4 (6.2 to 22.6)	NA	<.001
**Compliance for each patient presentation during total hospitalization with regard to no use**					
Chest radiography (for respiratory cause)	1726 (90)	1538 (85)	6.2 (0.5 to 11.9)	NA	.03
Albuterol	1800 (94)	1548 (86)	9.4 (5.6 to 13.2)	NA	<.001
Glucocorticoids	1877 (98)	1765 (98)	0.4 (−0.7 to 1.5)	NA	.50
Antibiotics (for respiratory cause)	1825 (95)	1677 (93)	2.9 (−0.8 to 6.6)	NA	.12
Epinephrine	1913 (100)	1805 (100)	NA	NA	NA
No. of medication doses during total hospitalization for those who received any medications, median (IQR)	3 (1 to 7)	3 (1 to 6)	NA	1.1 (0.7 to 1.6)	.78
No. of albuterol doses for those who received any albuterol, median (IQR)^c^	NA	NA	NA	NA	NA
First 24 h	2 (1 to 3)	2 (1 to 3)	NA	1.2 (0.9 to 1.6)	.29
During total hospitalization	2 (1 to 5)	2 (1 to 4)	NA	1.1 (0.7 to 1.7)	.77
While in ED	2 (1 to 3)	2 (1 to 3)	NA	1.2 (0.8 to 1.6)	.29
During inpatient treatment^b^	3 (1 to 9)	3 (1 to 8)	NA	1.0 (0.7 to 1.4)	.83
Length of stay, median (IQR), h	12 (2 to 42)	11 (2 to 45)	NA	0.9 (0.7 to 1.2)	.67
Admitted to hospital	1043 (54)	945 (52)	0.03 (−0.1 to 0.2)	NA	.62
ICU admission	63 (3)	41 (2)	0.4 (−0.2 to 1.0)	NA	.21

Abbreviations: ED, emergency department; ICU, intensive care unit; IQR, interquartile range; NA, not available.

^a^ Analyzed using model A, adjusted for stratification factors at randomization (country and on-site pediatric ICU).

^b^ Includes ICU.

^c^ Inhaled, nebulized, or oral doses.

No statistically significant difference between groups was observed for length of hospital stay (median intervention group, 12 [IQR, 2-42] hours; control group, 11 [IQR, 2-45] hours; IRR, 0.9; 95% CI, 0.7-1.2; *P* = .67) or ICU admission (intervention group, 63 [3%]; control group, 41 [2%]; RD, 0.4%; 95% CI, −0.2% to 1.0%; *P* = .21) (Table 4). There were no deaths in either group.

No statistically significant difference between groups was observed in the median number of medication doses during total hospitalization for infants who received any medications (intervention group median, 3 [IQR, 1-7]; control group median, 3 [IQR, 1-6] doses; IRR, 1.1; 95% CI, 0.7-1.6; *P* = .78). Similarly, no statistically significant difference between groups was observed in the median number of albuterol doses at any time point or from any managing team in infants who received any albuterol (Table 4).

### Hospital Compliance With Interventions

All 13 intervention hospitals undertook the majority of the 6 intervention components as per the study protocol. Total intervention fidelity scores (site protocol compliance) varied from 55% to 98% (mean [SD], 78% [13%]). Twelve (92%) of the 13 hospitals identified a medical and nursing clinical lead for the study from both the ED and pediatric inpatient teams, with all remaining engaged for the duration of the implementation period. Forty-seven (90%) of the 52 clinical leads attended stakeholder meetings, and 42 (81%) of clinical leads attended the train-the-trainer workshop, with at least 1 from each hospital attending. Clinical leads were requested to train 80% of staff with the intervention PowerPoint presentation within the first month. Five hospitals (38%) achieved this objective, 5 hospitals (38%) trained more than 50% of staff, and 3 hospitals (23%) trained less than 50% of staff within the required time frame. All hospitals continued regular staff training throughout the implementation period. Other educational materials provided were used at all hospitals, ranging from 40% to 90%. Seven audit and feedback cycles (100%) were completed by all hospitals, with results disseminated to staff.

## Discussion

Our results show that targeted interventions designed to address factors influencing bronchiolitis management can improve the care delivered to infants with bronchiolitis and deimplement unnecessary care. Specifically, we found a 14.1% difference in rates of compliance during the first 24 hours of hospitalization, favoring the intervention group for all 5 bronchiolitis guideline recommendations, with the greatest change seen in albuterol and CR use. Improvement occurred for management within ED visits, as inpatients, and throughout total hospitalization.

There is increasing awareness of the problem of overuse of low-value care in health care.^27,32^ Reducing the use of inappropriate health interventions is important for minimizing patient harm, maximizing resources, and improving evidence-based health care delivery.^33^ Despite the fact that deimplementation is generally harder than implementing new evidence, few frameworks exist to guide deimplementation, and there is no “magic bullet.”^27^ Our study is, to our knowledge, one of few using rigorous methods to show effectiveness in deimplementing low-value care and addressing the call to advance science in this field.^26,27^ Although only conducted within Australia and New Zealand, the robust design means outcomes are likely applicable to other developed countries. The greatest change was seen in increased albuterol and CR compliance with guideline recommendations, where previously this compliance had been problematic.^14^ Although some clinical situations may warrant CR use in bronchiolitis, routine use is not warranted and is associated with increased inappropriate antibiotic use.^14^ This topic has been the subject of many international Choosing Wisely campaigns.^32^ Therefore, it is acceptable that compliance with CR is close to 100%, whereas compliance with not using the pharmacotherapies should be 100%. Inappropriate use of pharmacotherapies is 4 to 7 times higher in Europe and North America compared with Australia and New Zealand, and albuterol use is the largest aspect of care requiring deimplementation, which emphasizes the generalizability of our study.^14^ Hospitals with lower baseline compliance than ours may see a greater effect size, particularly if drivers of inappropriate care are similar.

Previous studies to improve bronchiolitis care have used a quality improvement design using before-and-after evaluation of multifaceted interventions,^34,35,36,37,38^ with interventions developed by expert clinicians.^34,39,40^ These studies may have shown decreased use of inappropriate investigations and pharmacotherapies, but reliability is weakened by their nonexperimental design, which can be subject to major confounders. Although interventions developed by expert clinicians may by chance address factors influencing bronchiolitis management, they are not developed specifically to address identified factors influencing the staff delivering care, as in our study.^29^

Improving evidence-based bronchiolitis management and reducing exposure to low-value and potentially harmful therapies (important patient-centered outcomes for the infant and their family) was achieved without the unintended consequences of increased length of stay or ICU admissions. As length of stay is unchanged, cost savings will be limited to the cost (time and money) of the individual guideline recommendations that were deimplemented.

Our aim for 15% improvement after intervention was based on clinical importance, with power calculations assuming a conservative estimate of 50% compliance (local data showed 52% and 73% compliance).^14^ The final compliance difference of 14.1% improvement from a control group compliance of 73% was consistent with these assumptions and at the higher limit of improvements shown in implementation cluster RCTs.^41^ Although small secular trends of improved compliance occurred in the 3 years before the implementation year (eFigures 2 and 3 in Supplement 1), adjustment for these trends did not change the results (Table 3).

Intervention hospitals aimed to educate 80% of clinicians within the first month, as educating clinicians at the end of the bronchiolitis season would have had little effect on management. Only 38% of hospitals (n = 5) achieved this target, with a further 38% (n = 5) achieving greater than 50% education and education continuing throughout the intervention period in all hospitals. This outcome suggests that a lower target and continued education were effective. Whether compliance would have increased if all hospitals had achieved the original target is unknown. All hospitals completed the 7 audit and feedback cycles. Studies have demonstrated that showing clinicians their performance data compared with that of peers can be effective in improving guideline compliance.^42,43^ Clinical leads may have potentially valued this intervention favorably, with real-time data seen as important to monitor compliance and target areas of noncompliance. Furthermore, anonymously benchmarking each hospital against the top hospital may have encouraged improved compliance and competition. Feedback strategies to staff were designed to be multifaceted, with clinical leads disseminating results as locally appropriate. Importantly, all hospitals continued using all interventions throughout the implementation period. Given the complex nature of the intervention, undertaken without additional clinical lead time or financial input, a mean overall intervention fidelity score of 78% from a pragmatic trial design represents robust intervention use and reinforces the acceptability of interventions. Feedback on interventions from clinical leads was positive.

### Strengths and Limitations

Our study has potential limitations. Hospitals needed to have more than 135 bronchiolitis presentations per year to be eligible. Therefore, translating results to smaller or resource-limited hospitals needs to be considered cautiously. The study was only conducted within Australia and New Zealand. However, the 26 hospitals represented a diverse range of health care systems from the 2 countries and were comparable to the types of hospitals where children are managed across Australia and New Zealand.^44^ Hospital heterogeneity, with varying involvement of ED and pediatric inpatient staff in bronchiolitis management, is closely representative of hospitals throughout the developed world, likely making results broadly generalizable. Data collection was retrospective and subject to information bias due to missing data. However, this bias is likely minimal because data concerning pharmacotherapies and CR are well recorded in medical records. Furthermore, prospectively collecting data at control hospitals was inappropriate because it would introduce risk of the Hawthorne effect. Patient eligibility required both an ED and final diagnosis of bronchiolitis for inclusion. This requirement was potentially subject to selection bias; eg, if a patient with true bronchiolitis underwent a CR that was misinterpreted as bronchopneumonia, the different discharge diagnosis would result in the case being ineligible for study inclusion. Thus, true compliance may be lower than reported. No prospective measurement was made on control hospital dissemination activities, unlike intervention hospital dissemination activities, again owing to the possibility of the Hawthorne effect. Finally, no hospital was 100% compliant with all intervention components. All activity occurred within local clinical leads’ nonclinical time and existing local educational programs. Despite these limitations, this pragmatic design is likely to make the improvements seen generalizable. Sustaining practice change is challenging within health care settings, and there are limited postimplementation studies describing this process. Sustainability of practice improvement beyond the intervention period is unknown, and further follow-up of hospitals is required.

## Conclusions

The targeted interventions in this cluster RCT were effective in improving the treatment of infants with bronchiolitis without any identified negative consequences. Our deimplementation study followed a stepped process to identify who needed to do what differently. Our study first evaluated, then targeted, the drivers and behaviors of non–evidence-based practice, with behavior change techniques likely to change practice. We then evaluated these interventions in a cluster RCT. These results provide clinicians and hospitals with clear implementation strategies to address unnecessary treatment of infants with bronchiolitis.

## Notes

Supplement 1. **eAppendix.** Bronchiolitis Intervention Detail Based on TIDieR (Template for Intervention Description and Replication) **eTable 1.** Five Key Clinical Recommendations From the American Academy of Pediatrics and Australasian Bronchiolitis Guidelines **eTable 2.** International Classification of Diseases Codes Used for Identifying Patients for Data Collection **eFigure 1.** Process Used for Development of Targeted Interventions **eFigure 2.** Control and Intervention Group Compliance for Five Guideline Recommendations and Total Compliance (All) by Year **eFigure 3.** Individual Control and Intervention Hospital Compliance by Year
eReferences

Supplement 2.
Trial Protocol

Supplement 3.
Statistical Analysis Plan

Supplement 4.
Data Sharing Statement
