# Supplementary material for: Effectiveness of Targeted Interventions on Treatment of Infants With Bronchiolitis: A Randomized Clinical Trial
Source: JAMA Pediatr. 2021 Apr 12;175(8):797–806. doi: 10.1001/jamapediatrics.2021.0295 (PMC8042564; doi:10.1001/jamapediatrics.2021.0295)
Supplement: Supplement 1. — eAppendix. Bronchiolitis Intervention Detail Based on TIDieR (Template for Intervention Description and Replication) eTable 1. Five Key Clinical Recommendations From the American Academy of Pediatrics and Australasian Bronchiolitis Guidelines eTable 2. International Classification of Diseases Codes Used for Identifying Patients for Data Collection eFigure 1. Process Used for Development of Targeted Interventions eFigure 2. Control and Intervention Group Compliance for Five Guideline Recommendations and Total Compliance (All) by Year eFigure 3. Individual Control and Intervention Hospital Compliance by Year eReferences [file jamapediatr-e210295-s001.pdf]

Supplementary Online Content

Haskell L, Tavender EJ, Wilson CL, et al; the PREDICT Network. Effectiveness of targeted interventions on treatment of infants with bronchiolitis: a randomized clinical trial. *JAMA Pediatr*. Published online April 12, 2021. doi:10.1001/jamapediatrics.2021.0295

eAppendix. Bronchiolitis Intervention Detail Based on TIDieR (Template for Intervention Description and Replication)

eTable 1. Five Key Clinical Recommendations From the American Academy of Pediatrics and Australasian Bronchiolitis Guidelines

eTable 2. International Classification of Diseases Codes Used for Identifying Patients for Data Collection

eFigure 1. Process Used for Development of Targeted Interventions

eFigure 2. Control and Intervention Group Compliance for Five Guideline Recommendations and Total Compliance (All) by Year

eFigure 3. Individual Control and Intervention Hospital Compliance by Year

eReferences

This supplementary material has been provided by the authors to give readers additional information about their work.

© 2021 Haskell L et al. *JAMA Pediatrics.*

eAppendix. Bronchiolitis Intervention Detail Based on TIDieR (Template for Intervention Description and Replication)

1. Name

Bronchiolitis interventions.

2. Why

To improve the evidence-based management of infants with bronchiolitis by reducing the unnecessary use of chest x-ray (CXR), albuterol, glucocorticoids, antibiotics and epinephrine.

3. What

Educational intervention material

- **Australasian Bronchiolitis Guideline** in electronic and hard copy format (in full and bedside clinical versions). Rationale: Improve knowledge; change beliefs; improve confidence.
- **PowerPoint presentation covering:**
  - Overview of bronchiolitis.
  - The Australasian Bronchiolitis Guideline and key supporting evidence.
  - Data demonstrating international and local variation in bronchiolitis management.
  - Each of the five evidence-based recommendations from the Australasian Bronchiolitis Guideline with key messages to be delivered to clinicians (presentation utilized behavior change techniques most likely to effect change, having been identified from qualitative clinician interviews analyzed using the Theoretical Domains Framework (TDF)).
  - Supplementary PowerPoint slides providing additional evidence supporting guideline recommendations.

  Rationale: Improve knowledge; increase skills; change beliefs; feedback on performance; address barriers and enablers to evidence-based management; reinforce importance of evidence-based management and consequences of not following recommendations; positive reinforcement.

Additional educational materials

- **Clinician training video:**
  - Role modelling how to discuss bronchiolitis and its management with family.

  Rationale: Demonstrate/role model clinician behavior; increase skill; provide motivation.
- **Evidence fact sheets:**
  - Providing detailed evidence supporting no use of CXR, albuterol and antibiotics.

  Rationale: Improve knowledge; change beliefs.
- **Promotional materials:**
  - Poster promoting the Australasian Bronchiolitis Guideline and five key guideline recommendations.
  - Poster presenting data illustrating variation in evidence-based management of infants with bronchiolitis.

  Rationale: Reminder/prompt of recommended management; feedback on performance; provide motivation.
- **Parent/caregiver bronchiolitis information sheet:**
  - Providing information on bronchiolitis, expected illness trajectory, supportive care and guidance for when to seek medical review.

  Rationale: Improve knowledge; increase skill and confidence; provide encouragement and support.

4. What (procedure)

- **Clinician leads:**
  - Four clinical leads, one nursing and one medical lead from each of the emergency department (ED) and pediatric inpatient teams for the duration of implementation period.
  - Key tasks included:
    - Attend train-the-trainer one day workshop.

© 2021 Haskell L et al. *JAMA Pediatrics.*

        - Lead delivery of educational intervention and additional educational materials to clinicians.
        - Oversee completion of monthly audit and feedback.
        - Co-ordinate interventions over implementation period.

  Rationale: Provide consistent credible, influential and trustworthy leadership; increase knowledge and skills through education, influence and persuasion; clinical leads ensured interdisciplinary and interdepartmental coverage.

- **Stakeholder meeting:**
    - Aimed to gain site buy-in at an organizational, senior leadership and senior clinician level.
    - Study team met with four clinical leads, and key medical and nursing stakeholders at each site at the beginning of the study to discuss:
        - The Australasian Bronchiolitis Guideline
        - Data demonstrating international and local variation in bronchiolitis management.
        - Review and discussed the site's audit of compliance with five key bronchiolitis recommendations.
        - Discuss any anticipated local barriers to the interventions.

  Rationale: Create site buy-in; provide feedback on current management; knowledge of own practice variation is likely to drive change; increase knowledge of intervention process; identify and address any potential barriers.

- **Train-the-trainer workshop:**
    - One day workshop for all four clinical leads (one nursing and one medical lead from each of the ED and pediatric inpatient team).
    - Workshop content included:
        - The Australasian Bronchiolitis Guideline and key supporting evidence.
        - Implementation science and implementation research.
        - Findings from qualitative study identifying factors influencing bronchiolitis management.
        - Rationale for interventions (detailed stepped process using TDF, mapping to behavior change techniques, and informed by systematic reviews of intervention components).
        - Role modelling of how interventions were to be delivered.
        - Discussing the responsibilities and importance of clinical lead.
        - Planning time for clinical leads from ED and pediatric inpatient area to work together (encouraging interdisciplinary and interdepartmental communication and relationships).

  Rationale: Improve knowledge; change beliefs; optimize professional interdisciplinary and interdepartmental relationships; motivate clinical leads as drivers of change.

- **Audit and feedback:**
    - Sites completed seven monthly audit cycles of 20 infants who presented during the previous month (the first 10 discharged from ED; the first 10 discharged from pediatric inpatient ward) for each month of the implementation period.
    - Each site received a monthly audit report providing tables and graphs detailing:
        - Their site's compliance in the management of infants with bronchiolitis for:
            1. Number of presentations which complied with all five guideline recommendations.
            2. Number of presentations which complied with each of the five individual guideline recommendations.
            3. Comparison of site's current and previous month's audit, and baseline audit (from stakeholder meeting) for 1 and 2.
            4. Their site benchmarked against the top performing site for 1, 2 and 3.
    - Audit reports disseminated by clinical leads in ED and pediatric inpatients to their staff in verbal and written format.
    - Clinical leads encouraged to use audits to action plan and set targets for improvement.

© 2021 Haskell L et al. *JAMA Pediatrics.*

Rationale: Provide real-time feedback on targeted behaviors; motivate by benchmarking; promote goal/target specific action planning to optimize on-going improvement; increase knowledge; change beliefs.

5. **Who provided**

- Train-the-trainer workshop facilitated and delivered by research team members (included senior nurses, doctors and implementation researcher) and clinical opinion leaders (experts in bronchiolitis management).
- Interventions delivered at sites by four clinical leads (medical and nursing, from ED and pediatric inpatient team) who had undergone training at the train-the-trainer workshop, or delivery overseen by clinical leads as appropriate.
- Clinical leads were selected by ED and pediatric inpatient areas – 'ideal characteristics' of a clinical lead were discussed with sites.

6. **How**

- Australasian Bronchiolitis Guideline in electronic and hard copy format (in full and bedside clinical versions) provided to ED and pediatric inpatient team to utilize and disseminate within their teams.
- PowerPoint presentation delivered face-to-face to all nursing and medical staff within ED and pediatric inpatient teams.
- Clinician video shown to nursing and medical staff within ED and pediatric inpatient teams.
- Promotional materials displayed in ED and pediatric inpatient wards to prompt memory of the guideline recommendations and highlight variation in bronchiolitis management.
- Audit results and feedback provided to nursing and medical staff within ED and pediatric inpatient teams in verbal and written form: one-one-one, in small or larger group meetings, via email, in departmental bulletins or quality updates. Action planning and target setting for improvement encouraged.
- Parent/caregiver bronchiolitis information sheet available for sites to use in ED and pediatric inpatient wards.

7. **Where**

- All interventions delivered and utilized in ED and pediatric inpatient wards.
- PowerPoint presentation delivered face-to-face to all nursing and medical staff within ED and pediatric inpatient teams during nursing and medical meetings, educational sessions, grand rounds, departmental daily huddles.

8. **When and how much**

- Implementation period was from May to November 2017 and included:
    - PowerPoint presentation (10-30 minutes duration) – clinical leads aimed to educate 80% of nursing and medical staff within their ED and pediatric inpatient teams in the first month, then on-going education to ensure all nursing and medical staff educated over the duration of the implementation period.
    - Additional educational materials utilized in ED and pediatric inpatient wards over the duration of the implementation period.
    - Audit and feedback – seven monthly audit reports completed with results disseminated by clinical leads to nursing and medical staff within ED and pediatric inpatient teams.

9. **Modifying**

- Educational intervention presentations were streamlined to meet local site scheduling constraints.
- Slight variation in materials was allowed, ensuring that five guideline recommendations and their key messages remained and were reinforced.
- Sites contextualized promotional material for their ED and pediatric inpatient departments.
- Parent/caregiver bronchiolitis information sheet could have site logo and contact details added.

10. **Modification**

- No modifications were made by the research team during the implementation period.

© 2021 Haskell L et al. *JAMA Pediatrics.*

11. How well (planned)

- Clinical leads from ED and inpatient pediatric teams maintained a log during the implementation period detailing:
  - Educational sessions completed, number and type of clinicians, which department, duration and materials utilised.
  - Audit and feedback dissemination – frequency, mode of dissemination, number and type of clinicians.
  - Promotional materials used and duration of use.

12. How well (actual)

- All sites had clinical leads attend the train-the-trainer workshop and for the duration of the implementation period.
- All sites delivered the interventions as per study protocol.
- 5/13 sites educated 80% of nursing and medical staff in the first month; all sites continued education over the duration of the implementation period.
- All sites completed all seven audits and disseminated to nursing and medical staff.

© 2021 Haskell L et al. *JAMA Pediatrics.*

eTable 1. Five Key Clinical Recommendations From the American Academy of Pediatrics and Australasian Bronchiolitis Guidelines

**Clinical intervention**	**AAP Bronchiolitis Guideline (USA), 2014**[1]		**Australasian Bronchiolitis Guideline, 2016**[2]	
	**Quality of evidence**[a]	**Recommendation**	**Quality of evidence**[b]	**Recommendation**
Albuterol	Strong	Do not administer to infants and children	Strong	Do not administer
Antibiotics	Strong	Do not administer (unless concomitant bacterial infection, or strong suspicion)	Conditional	Do not administer
Glucocorticoids	Strong	Do not administer to infants	Strong	Do not administer
Epinephrine	Strong	Do not use in infants and children	Strong	Do not administer
Chest x-ray	Moderate	Should not be obtained routinely	Conditional	Is not routinely indicated

AAP=American Academy of Pediatrics

[a]The AAP policy statement “Classifying Recommendations for Clinical Practice” was followed in designating levels of recommendation.

[b]Grading of Recommendations Assessment, Development and Evaluation methodology and National Health and Medical Research Council strength of recommendations classification systems were used to review literature.

© 2021 Haskell L et al. *JAMA Pediatrics.*

eTable 2. International Classification of Diseases Codes Used for Identifying Patients for Data Collection

ICD code	Description
**ICD-10**	
J21	Acute bronchiolitis
J21.0	Acute bronchiolitis due to respiratory syncytial virus
J21.1	Acute bronchiolitis due to human metapneumovirus
J21.8	Acute bronchiolitis due to other specified organisms
J21.9	Acute bronchiolitis, unspecified
**ICD-9**	
466.19	Acute bronchiolitis due to other infectious organisms

ICD=International Classification of Diseases

© 2021 Haskell L et al. *JAMA Pediatrics.*

eFigure 1. Process Used for Development of Targeted Interventions[3]

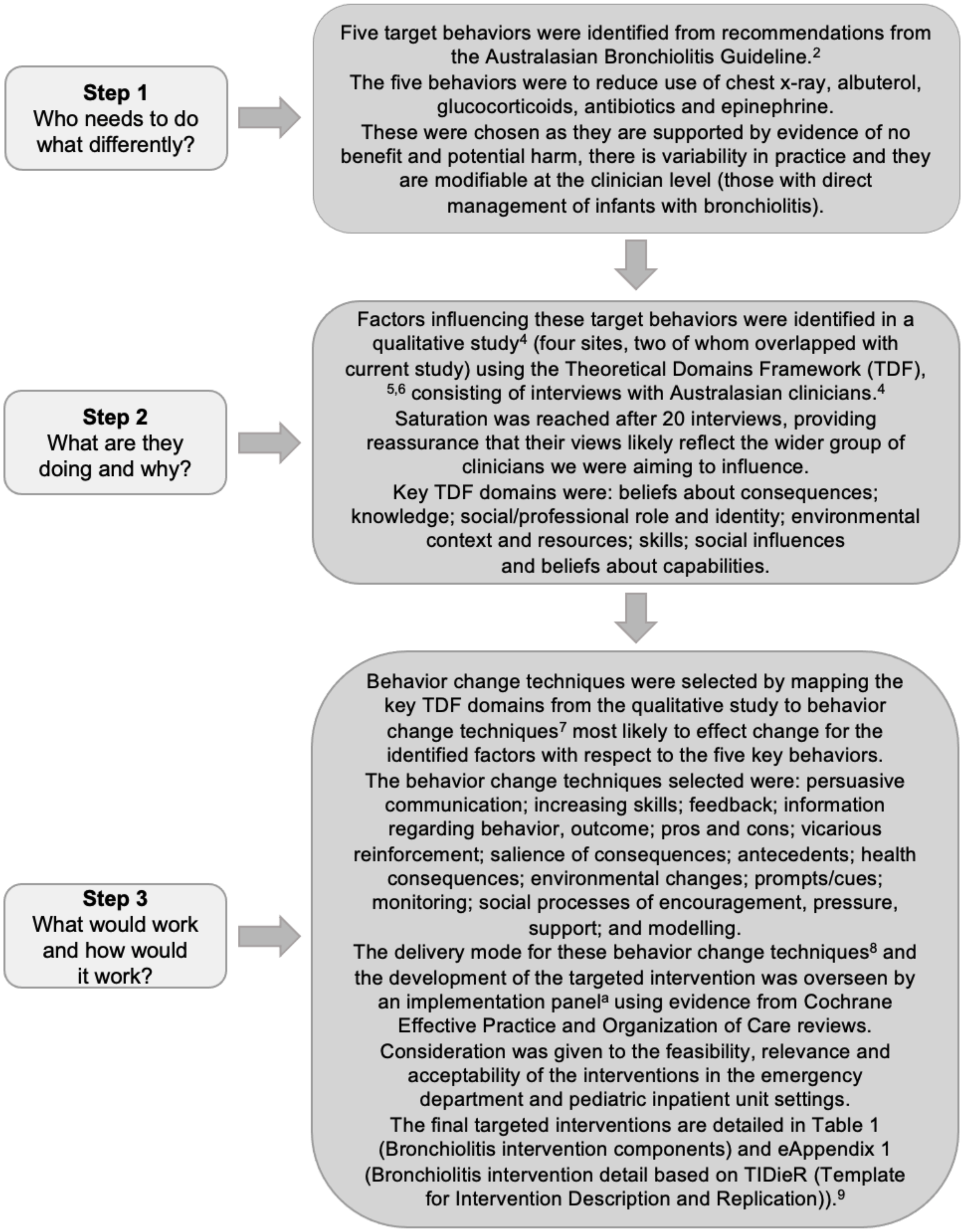

[a]Eight members including clinicians (senior nurses and doctors from emergency department and pediatric inpatient units from Australasia with direct responsibility for the management of infants with bronchiolitis) and an implementation scientist.

© 2021 Haskell L et al. *JAMA Pediatrics.*

eFigure 2. Control and Intervention Group Compliance for Five Guideline Recommendations and Total Compliance (All) by Year

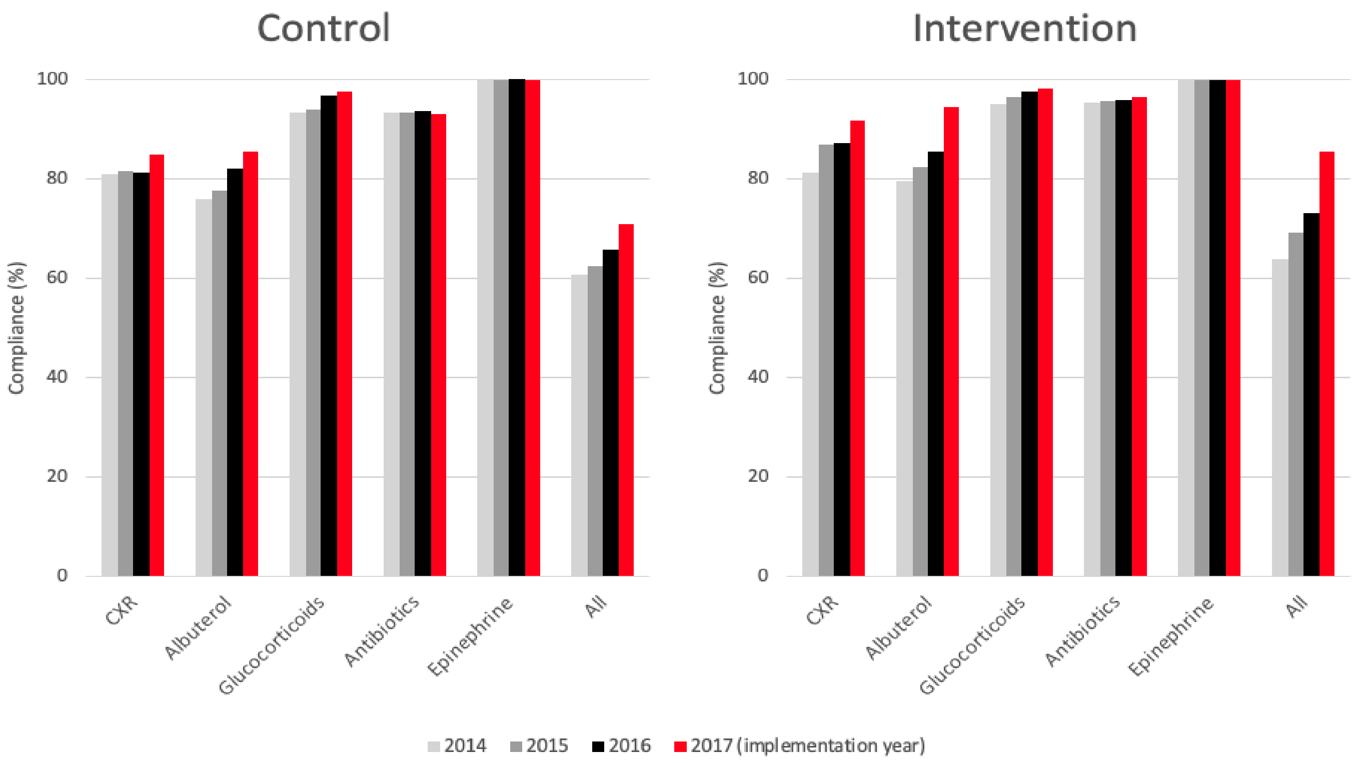

© 2021 Haskell L et al. *JAMA Pediatrics.*

eFigure 3. Individual Control and Intervention Hospital Compliance by Year

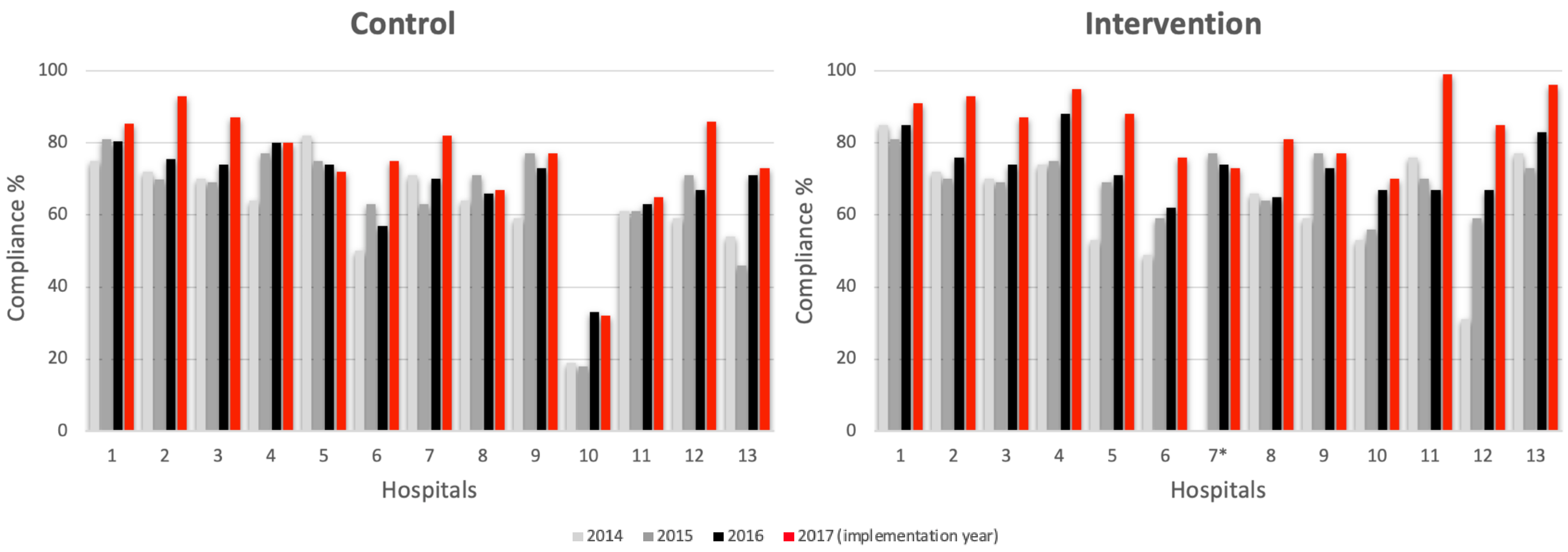

*Unable to collect 2014 data

© 2021 Haskell L et al. *JAMA Pediatrics.*

eReferences

1. Ralston SL, Lieberthal AS, Meissner HC, et al. Clinical practice guideline: the diagnosis, management, and prevention of bronchiolitis. *Pediatrics.* 2014;134(5):e1474-1502.
2. O'Brien S, Borland ML, Cotterell E, et al. Australasian Bronchiolitis Guideline. *J Paediatr Child Health.* 2018;55(1):42-53.
3. French S, Green S, O'Connor D, et al. Developing theory-informed behaviour change interventions to implement evidence in to practice: a systematic approach using the Theoretical Domains Framework. *Implement Sci.* 2012;7(38).
4. Haskell L, Tavender EJ, Wilson C, et al. Understanding factors that contribute to variations in bronchiolitis management in acute care settings: a qualitative study in Australia and New Zealand using the Theoretical Domains Framework. *BMC Pediatr.* 2020;20(1):189.
5. Michie S, Johnston M, Abraham C, et al. Making psychological theory useful for implementing evidence based practice: a consensus approach. *Qual Saf Health Care.* 2005;14(1):26-33.
6. Cane J, O'Connor D, Michie S. Validation of the theoretical domains framework for use in behaviour change and implementation research. *Implement Sci.* 2012;7(1):37.
7. Cane J, Richardson M, Johnston M, Ladha R, Michie S. From lists of behaviour change techniques (BCTs) to structured hierarchies: comparison of two methods of developing a hierarchy of BCTs. *Br J Health Psychol.* 2015;20(1):130-150.
8. Atkins L, Francis J, Islam R, et al. A guide to using the Theoretical Domains Framework of behaviour change to investigate implementation problems. *Implement Sci.* 2017;12(1).
9. Hoffmann TC, Glasziou PP, Boutron I, et al. Better reporting of interventions: template for intervention description and replication (TIDieR) checklist and guide. *BMJ.* 2014;348:g1687.

© 2021 Haskell L et al. *JAMA Pediatrics.*
