# Supplementary material for: Effectiveness of Targeted Interventions on Treatment of Infants With Bronchiolitis: A Randomized Clinical Trial
Source: JAMA Pediatr. 2021 Apr 12;175(8):797–806. doi: 10.1001/jamapediatrics.2021.0295 (PMC8042564; doi:10.1001/jamapediatrics.2021.0295)
Supplement: Supplement 2. — Trial Protocol [file jamapediatr-e210295-s002.pdf]

PROTOCOL

Knowledge Translation in Australasian Paediatric Acute Care Settings: a multi-centred, cluster-randomised controlled trial comparing a tailored, theory informed Knowledge Translation intervention versus passive dissemination of a bronchiolitis guideline. (PREDICT KT study)

CONFIDENTIAL

This document is confidential and the property of the *Paediatric Research in Emergency Departments International Collaborative (PREDICT).* No part of it may be transmitted, reproduced, published, or used without prior written authorisation from the institution.

Statement of Compliance

This document is a protocol for a research project. This study will be conducted in compliance with all stipulation of this protocol, the conditions of the ethics committee approval, the NHMRC National Statement on Ethical Conduct in Human Research (2007) and the Note for Guidance on Good Clinical Practice (CPMP/ICH-135/95).

INVESTIGATOR LIST

Name	Role
**Dr Stuart Dalziel**<br>**Starship Children’s Hospital**	**Co-Principal Investigator**
**A/Prof Ed Oakley**<br>**Murdoch Children’s Research Institute (MCRI) & Royal Children’s Hospital (RCH), Melbourne**	**Co-Principal Investigator**
**A/Prof Franz Babl**<br>**MCRI & RCH**	**Co-Investigator**
**A/Prof Meredith L Borland**<br>**Princess Margaret Hospital, Perth**	**Co-Investigator**
**A/Prof Elizabeth Cotterell**<br>**University of New England, Armidale & Armidale Rural Referral Hospital**	**Co-Investigator**
**Libby Haskell**<br>**Starship Children’s Hospital**	**Co-Investigator**
**Professor David Johnson**<br>**Alberta, Canada**	**Co-Investigator**
**A/Prof Nicolette Sheridan**<br>**University of Auckland**	**Co-Investigator**
**Francesca Orsini**<br>**MCRI**	**Statistician**
**Dr Emma Tavender**<br>**MCRI**	**Co-Investigator**
**Catherine Wilson**<br>**MCRI**	**Co-Investigator**
**Sharon O’Brien**<br>**PREDICT**	**Co-Investigator**

Contents

1. Protocol synopsis 5
2. Glossary of Abbreviations 12
3. Sponsor, funding and resources 13
   - 3.1 Sponsor details 13
   - 3.2 Funding and resources 13
4. Background and rationale 13
   - 4.1 Background 13
   - 4.2 The importance of Knowledge Translation in paediatric acute care 14
   - 4.3 Bronchiolitis clinical guideline development 15
   - 4.4 Key recommendations from guideline: 17

5 Research question 17

6. Study design 18
7. Objectives 20
   - 7.1 Primary objectives 20
   - 7.2 Secondary objectives 20
8. Outcomes 20
   - 8.1 Primary outcome 20
   - 8.2 Secondary outcomes 20

9 Process evaluation 21

10 Methodology 22
   - 10.1 Recruitment of hospitals and departmental agreement 22
   - 10.2 Recruitment of medical and nursing staff 23

11 Data collection and management 24
   - 11.1 Patient data 24
   - 11.2 Staff survey data collection and management 26
   - 11.3 Process evaluation data collection and management 26
   - 11.4 Economic evaluation 26
   - 11.5 Confidentiality 27

12 Statistical methods 27
   - 12.1 Sample size and calculations 27
   - 12.2 Staff survey data 28
   - 12.3 Process evaluation data 28

13 Proposed methods for protecting against sources of bias 28
13.1 Randomisation and masking of allocation 28
13.2 Cluster randomisation 28
13.3 Blinding 28
14 Details of Knowledge Translation intervention strategies 29
15 Ethics 31
15.1 Ethical approval and departmental agreement 31
15.2 Protocol Amendments 31
16 Protocol publishing 32
17 Reimbursement 32
18 Declaration of interests 32
19 Dissemination and authorship 32
20 Study management 33
20.1 Paediatric Research in Emergency Departments International Collaborative (PREDICT) 33
20.2 Study research group 33
20.3 Central study coordination 33
20.4 Study management – local 33

References........ 35

Appendices

1. Bronchiolitis guideline PICOT questions........ 36

2. Contact details of study management team........ 39

1. Protocol synopsis

TITLE	Knowledge Translation in Australasian Paediatric Acute Care Settings: a multi-centred, cluster randomised controlled trial comparing a tailored, theory informed Knowledge Translation intervention versus passive dissemination of a bronchiolitis guideline (PREDICT KT study).
STUDY DESIGN	Study type:<br><br>A multi-centre, cluster randomised controlled trial comparing a tailored, theory informed Knowledge Translation (KT) intervention versus passive dissemination of a bronchiolitis guideline.
PRIMARY OBJECTIVE	To determine the effectiveness of 3 months of tailored, theory informed KT intervention strategies versus passive dissemination of a bronchiolitis guideline in decreasing use of therapies known to have no benefit in infants with bronchiolitis.
SECONDARY OBJECTIVES	To evaluate differences in effectiveness of tailored, theory informed KT intervention strategies versus passive dissemination of a bronchiolitis guideline, at decreasing the duration of hospital stay for infants with bronchiolitis and to determine their relative effectiveness in tertiary paediatric hospitals and secondary paediatric hospitals.
OUTCOMES	The primary outcome is:<br><br>Compliance or non-compliance for each individual patient presentation with the guideline during the first 24 hours following presentation to the Emergency Department (ED) (acute care period) with regards to the use of key therapies / management processes known to have no benefit (chest x-ray, salbutamol, glucocorticoids, antibiotics, epinephrine).<br><br>The secondary outcomes are:<br><br>1. Compliance or non-compliance for each individual patient presentation with the guideline with regards to the use of key therapies / management processes known to have no benefit (chest x-ray, salbutamol, glucocorticoids, antibiotics, epinephrine):<br>a. While in ED<br>b. While an inpatient<br>c. During total hospitalization

	2. Compliance or non-compliance for each individual patient presentation with guideline recommendations during the first 24 hours following presentation to the ED (acute care period) with regards to the use of:<br>a. Chest x-ray<br>b. Salbutamol<br>c. Glucocorticoids<br>d. Antibiotics<br>e. Epinephrine<br><br>3. Compliance or non-compliance for each individual patient presentation with guideline recommendations during their total hospitalization with regards to use of:<br>a. Chest x-ray<br>b. Salbutamol<br>c. Glucocorticoids<br>d. Antibiotics<br>e. Epinephrine<br><br>4. Process evaluation including measure of receipt, delivery and acceptability.<br><br>5. Length of stay.<br><br>6. Death and or intensive care admission.<br><br>7. Health care costs (including cost associated with guideline development and implementation).<br><br>8. Median number of medication doses:<br>a. In acute care period<br>b. During total hospitalisation
PLANNED SAMPLE SIZE	Data has been utilised from the seven PREDICT sites that participated in the CRIB2 trial to determine power calculations.<br><br>A total of 1620 patients in each arm of the study (135 / site) will be required to detect a clinically meaningful absolute difference of 15% in the proportion of non-compliance patients between the two study groups (power of 80%, two-sided type I error level: 0.05).

STUDY POPULATION	24 hospitals across Australasia (including 6 New Zealand sites) each with an ED census of >135 cases of bronchiolitis per year will be randomly assigned to one of two interventions. Hospitals will be stratified by country (Australia, New Zealand) and the paediatric hospital status (tertiary or secondary providers of paediatric care).<br><br>All participating hospitals must fulfil all of the inclusion criteria and none of the exclusion criteria.<br><br>**Inclusion criteria for sites:**<br>To be eligible for this study, each hospital must:<br>• Have an ED census of >135 cases of bronchiolitis per year.<br>• Be willing to participate (either in the control or intervention arm).<br>• Have a signed departmental agreement from the ED and inpatient clinical directors.<br>• Have the ability to audit clinical notes (this will be tested during site visits with the principal investigator or designated study personnel).<br>• Nominate a clinical lead for medical and nursing for the duration of the study.<br>• Have the ability to recruit medical and nursing staff to complete staff surveys at baseline and endpoint.<br><br>**Exclusion criteria for sites:**<br>• Inability to audit clinical notes.<br>• Be averse to participating if randomised to the control arm (control sites will receive the KT training that intervention arm received, at the end of the study).<br>• Royal Children's Hospital, Melbourne; Princess Margaret, Perth; Starship Children's Health, Auckland. These sites have personnel who have significant lead roles in the study design and implementation or have been integral in the development of the bronchiolitis guideline. Excluding these hospitals will reduce the risk of potential bias in the study results.

	**<u>Identification of patients for audit</u>**<br><br>**Inclusion criteria of patients:**<br><br>1. Aged less than 12 months (at time of presentation), AND<br>2. A recorded diagnosis of bronchiolitis on discharge from ED to home, OR<br>3. A diagnosis of bronchiolitis on discharge from inpatient area AND a recorded diagnosis of bronchiolitis in ED<br><br>There is no exclusion on the basis of co-morbidities or transfer from other health care facilities. However, these two categories will be used for sub-group analyses.
STUDY DURATION	3 years 7 months data collection:<br><br>• 1/05/14 – 30/04/2016 - Retrospective chart audit (100 patients/ year) pre KT interventions (2 years)<br>• 1/5/16 – 30/4/17 – Retrospective chart audit (100 patients) - “washout period” during which bronchiolitis guideline is released (1 year)<br>• 1/05/17 – 30/11/17 – Retrospective chart audit (135 patients) post KT intervention (7 months)<br>• 1/1/18 – 30/11/18 – Study analysis and manuscript preparation<br><br>Sustainability of interventions will be assessed with a further chart audit of 135 patients per site from 1/05/18 – 30/04/19. This will be part of a separate study.
INTERVENTIONS	Interventions will be classified as tailored, theory informed KT intervention (intervention group) or passive dissemination (control group).<br><br>**Intervention group:** The tailored, theory informed KT intervention will be developed using a stepped approach of selecting and bringing together intervention components based on findings from semi-structured interviews (undertaken separately to this study, HREC 36179A), theory and evidence. This intervention will then follow a process of tailoring to site specific perceived barriers and enablers. The study’s KT implementation team will assist individual sites with tailoring KT interventions.

	**Control group:** Passive dissemination of guideline. An electronic and or printed copy of the Australasian bronchiolitis guideline will be sent to sites.<br><br>**Table 1** summarises the proposed components of the intervention. Only broad components are described in this protocol to reduce the likelihood of contamination of the control group. The process of developing the intervention with description of its content will be published in detail post data collection.
STUDY METHODOLOGY	**Recruitment**<br>The co-investigators will be responsible for approaching individual hospitals. Hospitals within the PREDICT network will be approached in the first instance to assess interest, willingness and ability to be involved. The remainder will be purposefully sampled to ensure adequate sampling across types and sizes of hospitals.<br><br>A total of 24 hospitals will be chosen to participate (including six New Zealand hospitals). Hospitals will be stratified by country and the classification of being tertiary or secondary providers of paediatric care.<br><br>This study will be unblinded.<br><br>Retrospective patient data extraction will be undertaken on clinical notes pre and post the release of the bronchiolitis guideline from all enrolled sites. A total of 3 years 7 months of data will be collected (100-135 patients/year/site as detailed previously) on relevant outcome measures: current interventions and length of hospital stay for bronchiolitis.<br><br>A further 7 months' patient data will be extracted retrospectively at 1-year post completion of KT intervention. This will provide data on sustainability of interventions. This will be undertaken as a separate study.
STATISTICAL METHODS	The primary analysis will be on an "intention to treat" basis.<br><br>The principal analysis will examine compliance or non-compliance for each individual patient with the guideline in the acute care period, ED and as an inpatient with regards to key therapies/management processes known to have no benefit.<br><br>A Generalized Linear Mixed Models (GLMM) will be used to estimate the marginal difference in the proportion of bronchiolitis patients treated in accordance with the existing guideline between the study arms. The GLMM approach will employ a logit link function and will include random effect terms for study site. Based on the GLMM, risk differences with 95%

	confidence internals will be computed. Missing outcome data will be imputed using multiple imputation models.<br><br>The details of the primary and secondary statistical analyses will be specified in a separate statistical analysis plan (SAP) which will be finalised before study data base lock. The SAP will detail covariates to be considered in the primary analysis model as well as subgroup and sensitivity analyses to be performed. The SAP will also outline the multiple imputation strategy to handle missing data.<br><br>An economic analysis will be carried out with costs associated to the payer (hospital). This will include: cost of ED presentation, cost of admission and cost of therapies/management known to be of no benefit. Additionally, the costs associated with the development of the Australasian Bronchiolitis Guideline and the KT intervention development and implementation will be analysed.<br><br>Process evaluation will occur to assess fidelity in the delivery of the KT interventions (what was delivered, to whom and how) as well as staff questionnaires. See section 9. Mixed methods will be used including qualitative interviews and quantitative surveys.

Table 1 Planned delivery of the intervention

Intervention and control group

- An electronic and or printed copy of the *Australasian Bronchiolitis Guideline* will be made available to all sites.

Intervention group only

- Face-to-face multidisciplinary key stakeholder meeting in each participating hospital with research group personnel to create buy-in at organisational level for the changes by discussing key findings from qualitative interviews and key recommendations from underlying evidence; discussing intervention components and how to overcome anticipated barriers in their implementation.

- Identification of up to four local champions (medical and nursing) from the ED and general paediatrics departments. Information on the types and characteristics of personnel suited to these roles will be provided to sites.

- A one-day train-the-trainer interactive workshop, led by content experts and the research team, attended by nursing and medical local champions. Will provide information and skills training in relation to KT, key-recommendations from the guideline and the role of the local champions in the study, process evaluation and data collection.

- Delivery of materials for local training in relation to key recommendations from bronchiolitis guideline. These will be delivered by local champions to their staff over a 3-month period. This will include: promotional material, reminders (electronic and/or paper based, stickers for notes), educational material, audit and feedback plan.

- Provision of relevant tools, materials, study manual and information books.

- Support for local champions via email, telephone or face-to-face during intervention period.

2. Glossary of Abbreviations

ABBREVIATION	TERM
ACEM	Australasian College of Emergency Medicine
AGREE	Appraisal of Guidelines for Research and Evaluation
AI	Associate Investigator
CI	Confidence Interval
CPI	Co- Principal Investigator
CRF	Case Report Form
CRIB STUDY	Comparative Rehydration in Bronchiolitis Study
CXR	Chest X-ray
ED	Emergency Department
EPOC	Effective Practice and Organisation of Care (Cochrane Group)
GCP	Good Clinical Practice
GRADE	Grading of Recommendations Assessment, Development and Evaluation
HREC	Human Research Ethics Committee
ITT	Intention to treat
KT	Knowledge translation
NEAF	National Ethics Application Form
NHMRC	National Health and Medical Research Council
PEM	Paediatric Emergency Medicine
PREDICT	Paediatric Research in Emergency Departments International Collaborative
PI	Principal Investigator
PICOT	Population Intervention Comparison Outcome Time
RACP	Royal Australasian College of Physicians
RCT	Randomised Controlled Trial
REDCap	Research Electronic Data Capture
RR	Relative Risk

3. Sponsor, funding and resources

3.1 Sponsor details

Study Sponsor	PREDICT
Contact name	Dr Stuart Dalziel
Address	Children's Emergency Department<br>Starship Children's Health<br>Auckland District Health Board<br>Private Bag 92024<br>Auckland 1142<br>New Zealand
Telephone	+64 9 3074949 x24225 / 021 869068
Email	sdalziel@adhb.govt.nz

3.2 Funding and resources

Funded by the National Health and Medical Research Council (NHMRC GNT1058560) Australia and the Health Research Council New Zealand (HRC 13/556). The funders have no role in study design, collection, management, analysis and interpretation of data; writing of the report and decision to submit for publication.

4. Background and rationale

4.1 Background

Knowledge Translation (KT) in emergency medicine is a relatively new field, and in paediatric emergency medicine (PEM) and paediatric acute care it is in its infancy. There are a number of ongoing trials assessing the effectiveness of various strategies for KT in emergency medicine and in PEM, although none of these are in Australasia.[1 2] PEM and acute care in different environments and countries has its own unique structure and inherent barriers to implementing practice change, thus KT strategies used in one country need to be reassessed during implementation in another to ensure optimal KT practices are found for the local environment. In order to establish optimal KT practices for local paediatric acute care environments, we plan to undertake a cluster randomised trial of KT implementation methods in Australia and New Zealand paediatric acute care, using bronchiolitis management as the basis for the implementation.

While there are differences in the health care systems of Australia and New Zealand, at a post-graduate educational level the two countries share common structures; The Australasian College of Emergency Medicine (ACEM) and the Royal Australasian College of Physicians (RACP) are responsible for training Emergency Physicians and Paediatricians, respectively. Trainees in both countries use the same curriculum and frequently work in both systems. PREDICT is an established research network for PEM across both Australia and New Zealand and involves both medical and nursing. Thus it is logical to conduct a study of KT implementation in hospitals from both countries, with a paediatric condition, bronchiolitis which requires care and input from both medical and nursing healthcare members.

Bronchiolitis is a good choice for testing KT implementation strategies for a number of reasons; 1. It is an extremely common disease that is seen in small rural hospitals as well as in tertiary centres;[3-5] 2. It is the most common reason to be admitted into hospital for infants aged <1 year in New Zealand, with >70 admissions/1,000 infants. Māori (relative risk (RR) 3.0), Pacific (RR 4.3), and those living in the most deprived quintile (RR 4.7) are at most risk;[6] Bronchiolitis accounted for 56 per cent of all admissions to Australian hospitals of infants aged less than one year in 2000-2001;[7] 3. Hospitalisation is the primary determinant of health care expenditures for the disease; 4. Management is well defined; [8,9] and necessitates the involvement of medical and nursing input. 5. Despite this, substantial variations in practice occur.[10] Therefore effective implementation strategies should lower overall health care interventions, improve patient care and allow redirection of healthcare funds to other areas.

The PREDICT network is well placed to undertake this study having completed a multi-centre RCT of intravenous versus nasogastric fluid replacement in children admitted with bronchiolitis (known as the CRIB study).[11] As part of this trial, data were collected on >3,000 admissions for bronchiolitis, over 3 years from seven Australasian sites (known as the CRIB2 study). These data show that interventions for which there is high-level evidence suggesting that they are ineffective, were used at least once in 27% to 48% of bronchiolitis admissions (unpublished data, Ineffective interventions included inhaled salbutamol, inhaled epinephrine, oral glucocorticoids, chest x-ray and antibiotics).

The research project has involved the development of an Australasian clinical guideline for the management of bronchiolitis. This guideline will be implemented using different strategies via the KT project. It is recognised that in order to effectively manage conditions there needs to be agreement with the process from other specialty groups within individual hospitals. Thus the guideline developed as part of this project, will be for both ED and in-patient management of infants with bronchiolitis.

4.2 The importance of Knowledge Translation in paediatric acute care

Improving health care delivery requires both increase in the quality of care that is received and decrease in the exposure to unnecessary care-related events (potential adverse events). Internationally, there is a strong body of evidence which shows that health care delivery, both

medical and nursing, fails to use evidence optimally. There is still a significant gap between what clinicians know and what they do. As a result suboptimal quality of care is received, exposure to unnecessary care-related events occurs at a high frequency, with both potentially resulting in reduced quality of life or even avoidable loss of life. Ongoing investment in clinical research is timely, costly and ultimately only significant if newfound knowledge is reflected in clinical practice resulting in improvement in cost efficient, high quality health care and best outcomes for children and families. Despite knowing this, the gap between evidence and practice is not disappearing.

Understanding the most effective and efficient means of translating evidence into practice needs to be fully understood and this new knowledge utilised to reduce evidence–practice gap. It is now accepted that simply providing evidence from clinical research is insufficient for the provision of optimal care. For optimal care to occur research dissemination into everyday practice needs to occur. Recognition of this critical issue has resulted in the creation of a specific area of practice called KT: the methods needed for closing gaps from existing knowledge to optimal practice. Despite there being more than 25 years of knowledge synthesis activity in health care aiming to ensure access and uptake of new evidence, positivism remains that change in clinically important practise can be achieved with current KT strategies.[12]

KT in paediatric nursing and medicine is a relatively new field, and in PEM, is in its infancy. Approaches to KT are varied and it is clear that barriers and challenges experienced in one region or country are not able to be accurately translated to a different context. Therefore it is difficult and, to an extent inappropriate to apply knowledge obtained from studies in other countries to our unique health care structure and environment. This study will contextualise KT intervention strategies to the localised environments of Australian and New Zealand EDs and inpatient settings.

Studies assessing the effectiveness of KT implementation strategies have found variable, inconsistent results. Cochrane EPOC systematic reviews have shown that interventions consistently showing effectiveness include interactive educational meetings, educational outreach visits, reminders (either manual or computerised) and multifaceted interventions (defined as a minimum of two combined interventions). Interventions that have shown some improvement include audit and feedback, the use of local champions, local consensus processes and patient-mediated interventions. Interventions that consistently show little or no effect are didactic educational meetings and educational materials such as those distributed for recommendations of clinical care, including practice guidelines, electronic publications and audio-visual materials. [13,14]

4.3 Bronchiolitis clinical guideline development

Viral bronchiolitis is the commonest lower respiratory tract infection in infants less than 12 months of age and is the most frequent cause of hospitalisation in infants under 6 months of age.[15,16] As discussed earlier, research conducted by the PREDICT network has identified

that there is substantial variation in practice patterns in Australasia in relation to the management of bronchiolitis. This is despite treatment being well defined with supporting high quality Cochrane meta-analysis evidence.

Guidelines can be formally defined as “systematically developed statements to assist practitioner and patient decisions about appropriate care for specific clinical circumstances”.[17] In order to align and improve Australasian practice with the evidence, a high quality, evidence based, and consensus lead guideline for the management of bronchiolitis in infants presenting to and admitted into hospitals has been developed. This guideline is the first national, or binational, guideline in acute paediatric care for Australia and New Zealand. Formulation of this guideline has followed international best practice standards for guideline development using AGREEII, GRADE and NHMRC standards:

1. Identification of key PICOT research questions (see **appendix 1**).
2. Systematic literature search of multiple electronic databases including Ovid Medline, Ovid Embase, PubMed, Cochrane Review library and Cochrane library for systematic reviews and randomised controlled trials in addition to searches for reviews/ meta analyses / other guidelines on bronchiolitis and across the relevant question areas. PICOT questions not well supported by systematic reviews and randomised controlled trials have been searched for non-experimental observational evidence (e.g. case control studies and cohort studies).
3. Evidence tables and summaries of evidence (GRADE and NHMRC) prepared for each question.
4. Evidence based recommendations developed for those questions with high quality supportive evidence. These will be the focus of this study.
5. Consensus clinical care statements developed for those questions with low quality supportive evidence.
6. The above processes have led to a series of statements which provide a bedside guideline which is succinct and user friendly. There is also a separate document with references and evidence to support the guideline.
7. The guideline includes flow diagrams of patient care for infants with bronchiolitis, high-lighting key assessment and treatment points throughout the patient journey.
8. Consultation of the draft guideline includes; Australian Paediatric Society, New Zealand Paediatric Society, Australasian College of Emergency Medicine, Royal Australasian College of Physicians, New Zealand Emergency Medicine Network, Australian and New Zealand Nursing Colleges and clinical leads of general paediatrics and emergency departments at tertiary paediatric hospitals.
9. Release of the final guideline will be following endorsement by key stake-holders.

A 22-person Guideline Working Group (under the guidance of a five-person Executive Group who provided expert advice during the development process) have undertaken the development of this guideline. Members of this group included emergency nurses, nurse practitioners, general paediatricians, emergency physicians, paediatric intensive care physicians, paediatric respiratory physicians and clinicians. Members were selected from

rural, secondary metropolitan and tertiary paediatric hospitals, with representation across Australia and New Zealand. This group has met face-to-face with resulting outputs being achieved through smaller working groups via teleconference.

4.4 Key recommendations from guideline:

Medication:

- Beta 2 agonists – Do not administer beta 2 agonists.
    - NHMRC strength of recommendation – **A**
    - GRADE quality of evidence – **Strong**
- Corticosteroids – Do not administer systemic or local glucocorticoids (nebulised, oral, intramuscular (IM) or IV).
    - NHMRC strength of recommendation – **B**
    - GRADE quality of evidence - **Strong**
- Adrenaline – Do not administer adrenaline (nebulised, IM or IV).
    - NHMRC strength of recommendation – **B**
    - GRADE quality of evidence - **Strong**
- Antibiotics – Do not use antibiotics to treat infants with bronchiolitis.
    - NHMRC strength of recommendation – **B**
    - GRADE quality of evidence – **Conditional**

Investigations:

- Chest x-ray (CXR) **-** Is not routinely indicated.
    - NHMRC strength of recommendation – **D**
    - GRADE quality of evidence – **Conditional**

This recommendation has been chosen as CXRs have been shown to have the potential to lead to unnecessary treatment with antibiotics with subsequent risk of adverse events.

5 Research question

This study aims to answer the following question: In infants (<1 year of age) presenting to EDs and admitted to inpatient settings with bronchiolitis in Australia and New Zealand, does a

tailored, theory informed KT intervention increase the uptake of key clinical recommendations from an Australasian bronchiolitis guideline in reducing the use of therapies/management known to be of no benefit compared to passive dissemination of the guideline?

6. Study design

This is a multi-centre, cluster-randomised controlled trial with the hospital being the cluster, including the ED and general paediatric staff members involved in the care of infants with bronchiolitis. A randomised design is advantageous in evaluating the effectiveness of an intervention since bias is minimised when estimating intervention effects compared with other study designs. Clusters have been chosen for the following two reasons: the intervention is targeted to the staff involved in the care of infants with bronchiolitis, and the hospitals represent patient populations in geographical areas, removing the use of an individually randomised design. [18,19]

See **figure 1** for the study process design.

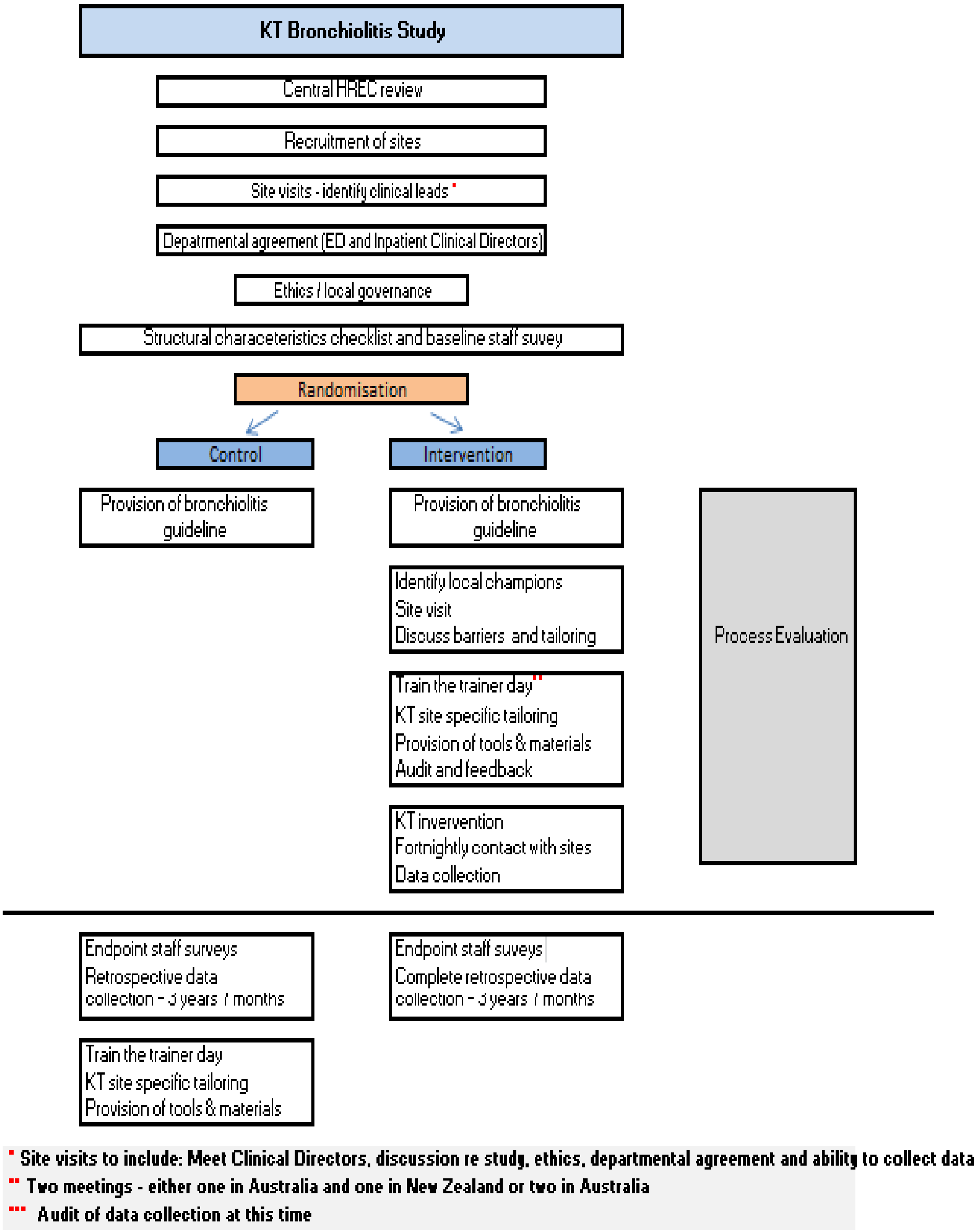

Figure 1 KT bronchiolitis study process design

7. Objectives

7.1 Primary objectives

To determine whether a tailored, theory informed KT intervention is effective in decreasing the use of therapies known to be of no benefit, compared to passive dissemination of a bronchiolitis guideline.

7.2 Secondary objectives

To evaluate differences in effectiveness of KT intervention strategies versus usual practice in decreasing the length of stay, cost of hospital stay for infants with bronchiolitis and to determine their relative effectiveness in hospitals of differing caseloads.

8. Outcomes

8.1 Primary outcome

Compliance or non-compliance for each patient presentation with the guideline during the first 24 hours following presentation to ED (acute care period), with regards to the use of key therapies/management processes known to have no benefit (chest x-ray, salbutamol, glucocorticoids, antibiotics, epinephrine).

8.2 Secondary outcomes

1. Compliance or non-compliance for each patient presentation with the guideline with regards to the use of key therapies / management processes known to have no benefit (chest x-ray, salbutamol, glucocorticoids, antibiotics, epinephrine):
    a. While in ED
    b. While an inpatient
    c. During total hospitalization

2. Compliance or non-compliance for each patient presentation with guideline recommendations during the first 24 hours following presentation to the ED (acute care period) with regards to the use of:
    a. Chest x-ray
    b. Salbutamol
    c. Glucocorticoids
    d. Antibiotics
    e. Epinephrine

3. Compliance or non-compliance for each patient presentation with guideline recommendations during their total hospitalization with regards to use of:
    a. Chest x-ray
    b. Salbutamol
    c. Glucocorticoids
    d. Antibiotics
    e. Epinephrine

4. Process evaluation including measure of receipt, delivery and acceptability.

5. Length of stay

6. Death and or intensive care admission

7. Health care costs (including cost associated with guideline development and implementation)

8. Median number of medication doses:
    a. In acute care period
    b. During total hospitalisation

9 Process evaluation

Process evaluation can provide insight into why an intervention was effective or why it was not. This provides better understanding of KT intervention effect and how it may have changed practise or why it didn't change practise. It is for this reason that process evaluation is included in this study design. Process evaluation will be used to assess fidelity (whether the intervention was successfully and consistently delivered as planned and to whom it was delivered), the quality of implementation, and to characterise contextual factors associated with variation in outcomes.

It is important that any variability in implementation (be it intended or unintended) be clearly reported with descriptions of interventions made explicit. This is in order to enable replication or omission of defined KT interventions in future planning of studies or quality improvement processes.

See **Table 2** for Intervention components and process evaluation methods

10 <u>Methodology</u>

10.1 Recruitment of hospitals and departmental agreement

Clinical directors of EDs and general paediatric inpatient settings within the PREDICT network will be approached by phone in the first instance by co-investigators. This is to assess interest, willingness and ability to be involved. The remainder of hospitals will be purposefully sampled through the Australasian Society for Emergency Medicine ED Directory list of 24 hour Australian EDs (regional and metropolitan) until a total of 24 hospitals have been recruited (including 6 New Zealand hospitals). This process will ensure selection across states and hospitals that provide different levels of paediatric care. Hospitals who have co-principal investigators in this study or who had significant involvement in the writing of the bronchiolitis guideline will be excluded to reduce the potential risk of bias in the study results.

Following an initial phone conversation, a recruitment pack detailing the proposed study will be sent to both nursing and medical ED and inpatient clinical directors. This pack will include an invitation letter from the research team, explanatory statement, and an expression of interest form. The clinical directors will be encouraged to discuss the trial with relevant personnel in their departments. Phone or email contact will be made by the research team approximately one week after sending the recruitment pack to check receipt and ascertain interest. A recruitment meeting will be organised (via telephone or face-to-face) to meet with relevant stakeholders in the ED and inpatient setting to discuss details and logistics of the study (with expectations of hospitals as well as the study team detailed). Hospitals will be included once a departmental agreement has been signed by both the ED and General Paediatric Clinical Directors (or equivalent). Hospitals will be excluded if they cannot get both Clinical Directors agreement to participate.

Inclusion criteria for hospitals:

- Have an ED census of >135 cases of bronchiolitis per year.
- Be willing to participate (either in the control or intervention arm).
- A signed department agreement by both ED and inpatient clinical directors.
- Have the ability to collect the required retrospective patient data from clinical notes.

Exclusion criteria for hospitals:

- Inability to audit clinical notes.
- Be averse to participating if randomised to the control arm.
- Royal Children's Hospital, Melbourne; Perth Children's Hospital (formerly known as Princess Margaret), Perth; Starship Children's Health, Auckland.

10.2 Recruitment of medical and nursing staff

Medical and nursing staff (up to 20 clinicians) from ED and general paediatrics areas in the intervention arm and control groups will be invited to complete two questionnaires (at baseline and endpoint). The aim of this is to explore factors that may influence how they manage infants with bronchiolitis. Staff participants will be selected from the active list of medical and nursing staff working in the area. Each staff member will be given a research ID and the central research team will select which IDs are to complete the survey. The site local champion will organise delivery of an invitation letter (which includes approval by the Clinical Director), and the survey to the selected staff members via mail or email. Consent will be implied if a completed survey is returned directly to the central research team. This will be stated clearly in the introduction to the questionnaire. Additional staff will be randomly selected to complete a survey if there are staff who are lost to follow-up.

Inclusion criteria for nurses are:

- Current ED or general paediatric employee
- On active practice roster
- Registered or enrolled nurse

Inclusion criteria for medical are:

- Current ED or general paediatric employee
- On active practice roster
- Registrars, House Officers (or equivalent) or consultants

Exclusion criteria for nursing and medical staff are:

- Students / interns
- Clinicians not currently engaged in clinical practice
- Agency or bank staff (nurses) or locums (medical)

All ED and general paediatric Clinical Directors (or their representative) will be invited to complete two checklists at baseline and endpoint by telephone regarding departmental characteristics e.g. medical and nursing staff numbers, annual ED / admission numbers, short stay facility.

11 Data collection and management

11.1 Patient data

Identification of patient records for data collection

In order to determine the effect of the intervention on clinical practice outcomes, data extraction from a random selection of patient records will be conducted by chart auditors appointed at each site.

Inclusion criteria:

1. Aged less than 12 months (at time of presentation), AND
2. A recorded diagnosis of bronchiolitis on discharge from ED to home, OR
3. A diagnosis of bronchiolitis on discharge from inpatient area AND a recorded diagnosis of bronchiolitis in ED

(There is no exclusion on the basis of co-morbidities, transfer from other health care facilities or representation with bronchiolitis. However, these two categories will be used for sub-group analyses).

The diagnosis of bronchiolitis will be initially identified by the admitting and discharge diagnosis. Trained site chart auditors will screen charts for eligibility with respect to the inclusion and exclusion criteria. A random number generator will generate a series of random numbers, corresponding to these charts, to identify those to be audited. Should there be an insufficient number of eligible charts identified from the random number sequence, further random numbers will be generated according to the aforementioned procedure, until the target sample size is reached at each centre.

Patient data to be collected

- Date of birth
- Sex
- Ethnicity
- Date and time of ED presentation
- Date and time of disposition from ED and where to – admitted or discharged home
- Date and time of discharge from inpatient setting
- Date and time of transfer to intensive care unit
- Date and time of transfer to ward from ICU
- Past history
- CXR during hospitalisation, time taken, CXR report
- Salbutamol administration during hospitalisation: number and timing of doses, discharged on salbutamol

- Adrenaline administration during hospitalisation: number and timing of doses
- Glucocorticoid administration during hospitalisation: number and timing of doses
- Antibiotics administration during hospitalisation: number and timing of doses
- Supplementary oxygen during hospitalisation
- High flow during hospitalisation

Patient data management responsibilities

Data collection and accurate documentation are the responsibility of the site study staff under the supervision of the site principal investigator.

Data collectors will be trained in auditing of clinical data with the aim of maximising consistency both in identification of patients and minimising selective auditing. They will enter data into the REDCap study database. Each site will be required to keep an electronic log book. This will contain a patient research identification code so that data is re-identifiable at the site if required e.g. if data queries arise.

Patient Data storage

A REDCap database will be used which is a web-based, secure, software solution to data management and workflow methodology, used extensively for managing multi-centre research data. Data collectors will receive a data collection manual with instructions (such as definitions and data dictionary). The REDCap database will be designed to minimise data entry errors such as the inability to leave particular fields open, or a warning sign when an answer is outside an expected range. Data may be recorded on hard copy clinical research forms (CRFs) and then entered in to REDCap or entered directly in to REDCap. Only nominated staff will have password protected site specific access. Sites will be able to view their own study data and only overall data administrators will be able to see coded data from all sites.
During site visits, a small sample (10-20) of clinical notes will be reviewed by both the study team and the local data collection personnel in order to formally test reliability and accuracy of data extraction.

Retrospective data collection will be undertaken on clinical notes pre and post the release of the bronchiolitis guideline from all enrolled sites. A total of 3 years 7 months of data will be collected (100 - 135 patients/year/site) on relevant outcome measures, therapies used, and length of hospital stay. This will include 3 years of data (100 patients per year) prior to intervention and then 7 months of data (135 patients) following the introduction of the guideline and intervention. There will be a further 7 months of data (135 patients) collected following this which will be used to assess sustainability of KT interventions.

Study data and records retention

All hard copy CRFs will be kept in a locked and secure location at study sites. These will be kept for 5 years or as per local policy.

11.2 Staff survey data collection and management

Identification of staff

Recruitment and consent of staff for baseline and endpoint surveys has previously been detailed in section 10.2.

Staff survey management

Unidentified, completed surveys, with a site code only, will be returned directly to the researchers either be email to the research staff or posted in a self-addressed envelope. Data will be entered into the database. Local champions and data entry staff at each site will not have access to the data. Researchers will have access to the data and study participant numbers, but not identifiable information other than the site code.

Staff survey record retention

Anonymous staff interview data will be kept in a locked and secure location in MCRI, Melbourne for 5 years following completion of the study.

11.3 Process evaluation data collection and management

Process evaluation data storage

Part of the role of the local champion (or their equivalent) in the intervention arm, will be to ensure data is collected on KT intervention delivery and entered into REDCap e.g. educational sessions undertaken including numbers of attendees (medical and nursing), audit and feedback undertaken and how this was presented to staff. Staff at each site will not see or have access to any other site's information. The research team will be able to access this information including frequency of logging on to REDCap to record interventions, chart audits and resultant audit reports being run.

During the KT intervention period, the research team member responsible for each site will record frequency of emails, phone calls and face-to-face meetings with local opinion leaders at each site and also record these on REDCap.

Process evaluation data management

The process evaluation data stored on REDCap will be kept for a minimum of 5 years.

11.4 Economic evaluation

The costs and benefits associated with compliance to the guideline are clearly relevant to the question: what is the cost-effectiveness of an implementation intervention to increase compliance to the five key recommendations from the guideline? The economic evaluation in this study will look at the trade-off between the increase in compliance to the guideline regarding the management of infants with bronchiolitis and the additional costs (savings)

arising from the implementation intervention. The evaluation will also take in to account the cost of the development of the Australasian bronchiolitis guideline. A Health Economist will be sought to undertake this evaluation.

11.5 Confidentiality

Confidentiality is strictly held in trust by the participating investigators, research staff, and the hospitals. This confidentiality covers all information collected from participating hospitals and their staff. The study protocol, documentation, data and all other information generated will be held in strict confidence.

No information concerning the study or the participant data will be released to any unauthorized third party, without prior written approval of the sponsoring institution. Authorized representatives of the sponsoring institution may inspect all documents and records required to be maintained by the Investigator, including but not limited to, medical records. The local study site will permit access to such records. All evaluation forms, reports and other records that leave the site will be identified only by the unique participant identification number to maintain participant confidentiality with originals kept at the sites in locked cabinets only accessible to research staff. Information will not be released without written permission of the hospital, except as necessary for monitoring by HDEC or regulatory agencies.

12 Statistical methods

12.1 Sample size and calculations

The primary outcome of this study is correct use of a bronchiolitis guideline in reducing therapies/management known to have no benefit (CXR, salbutamol, adrenaline, antibiotics and glucocorticoids). In the calculation of sample size for this outcome, adjustment has been made for the clustered nature of the design. The aim is to power the study to show an absolute decrease in assignment of intervention/therapies known to be of no benefit of at least 15%. The rationale for selecting this difference is based on justifying the resource intensive intervention.

Data from the seven PREDICT sites that participated in the CRIB2 trial (preliminary data on inappropriate interventions: CXR performed, salbutamol given, oral or IV steroids given, antibiotics given, adrenaline given) were used to determine power calculations for this study. This showed that the proportion of individuals who received at least one inappropriate intervention, ranged from 0.36 to 0.61 with a pooled estimate of about 52% [confidence interval based on a random effects model: 0.45 to 0.59].

The sample size calculations took the nature of the outcome (binary variable outcome variable: guideline-conform treatment yes/no) as well as the issue of clustering (heterogeneity in estimated proportions between sites that exceeds variability being explained by random sampling) into account.

The power calculations revealed a necessary group sample size of 1620 individuals, assuming an average cluster size of 135 patients (24 sites). These sample size calculations assumed (informed by preliminary data), an intra-cluster correlation coefficient of <0.06 as well as a proportion relative frequency of the primary outcome of 50% in the control arm.

12.2 Staff survey data

Data from the staff surveys will be coded and analysed using descriptive statistics to obtain frequencies, means and standard deviations for variables of interest. Means scores will be compared pre and post intervention to determine change.

12.3 Process evaluation data

Process data will be analysed using descriptive statistics to obtain frequencies, means and standard deviations for variables of interest.

13 Proposed methods for protecting against sources of bias

13.1 Randomisation and masking of allocation

Randomisation of hospitals within both strata (by country as well as tertiary or secondary hospital) into either KT intervention or control group will be completed using randomisation computer software, by a statistician who is not affiliated with the study. Additionally, a random sample of patient notes will also be undertaken as detailed in section 11.1.

13.2 Cluster randomisation

This study specifically focuses on health care personnel (medical and nurses). A potential threat to the validity of this study is if health care personnel from the KT intervention arms and control communicate. The researchers will aim to minimise contamination. The majority of recruited hospitals will be in different regions around Australasia, with significant physical distance between them which will limit some contact between sites.

13.3 Blinding

Due to the nature of the intervention, it will not be possible to blind staff members involved in the study to group allocation. This is a potential threat to the validity of the study. As mentioned previously, communication between intervention and control groups will not be actively encouraged but still may occur.

Ideally hospitals will use local data collectors not associated with patient care in ED or the general paediatric inpatient areas and who are not aware of study outcomes. A percentage

of data collection at each site will be independently audited to confirm the authenticity of the data. The data collection training will focus on the operational aspects of the study.

14 Overview of Knowledge Translation intervention strategies

Ensuring that KT intervention strategies are successful, practicable and sustainable in the reality of a practice setting is imperative. Additionally, being mindful of working within both the current hospital resources and the reality of the healthcare setting is vital.

Intervention arm:

- Tailored, theory informed intervention built upon by qualitative interviews (from a previous study; HREC 36179A) from a range of hospital types and medical and nursing personnel to determine barriers and enablers in implementing guideline. Intervention components will be tailored to target these identified barriers/enablers.
- A selection of KT interventions that individual hospitals assigned to this group will be assisted in choosing from. The study's KT implementation team will assist sites with tailoring KT interventions to their respective site following assessment of site specific perceived barriers and enablers. It is envisaged that the intervention will include the following core items: training of clinical leads, training of local staff by clinical leads, implementation of promotional material /reminders and audit and feedback. This will occur over an 11 month period, approximately.

Control arm:

- Passive dissemination: An electronic and or printed copy of the *Australasian Bronchiolitis Guideline* will be sent to all sites.

See **Table 1**: Planned delivery of the intervention.

See **Table 2:** Intervention components and process evaluation method.

Table 2 Intervention components and process evaluation method

<u>Intervention sites</u> – intervention includes delivery, content, receipt and acceptability

- *Local champions* – suitability, availability, perception of interventions
  - Evaluate through randomised questionnaires to nursing and medical staff and survey of all local champions.

- *Train the trainer day* – relevance and applicability, attendance, materials provided
  - Evaluate by questionnaires to attendees.

- Educational sessions / workshops:
  - Evaluate through number of nursing and medical staff attendances (from attendance record sheets); frequency of sessions (from attendance sheets); content of sessions through randomised questionnaires to nursing and medical staff and survey of all local champions and site specific log on REDCap (post intervention).

- Promotional material / reminders:
  - Evaluate through randomised questionnaires to nursing and medical staff and survey of all local champions (post intervention).
  - Evaluate through site specific log on REDCap (post intervention and during data collection).

- Audit and feedback:
  - Evaluate through randomised questionnaires to nursing and medical staff and survey of all local champions (post intervention).
  - Evaluate frequency this was undertaken through audit and reports generated from REDCap (post intervention and during data collection).
  - Evaluate through site specific log on to REDCap (post intervention and during data collection).

- Research group contact with sites:
  - Evaluate through log kept by site research team as to frequency of contact, how contacted and content of discussions (during intervention).
  - Evaluate through questionnaires of all local champions (post intervention).

<u>Control sites</u>

- *Intervention components utilised*
  - Evaluate through questionnaires to clinical directors on delivery of any intervention components e.g. was there a local champion / education provided to staff (7months post guideline release).

15 Ethics

15.1 Ethical approval and departmental agreement

Pre-recruitment ethical approval of this trial will be sought through a central National Ethics Application Form (NEAF). When participating hospitals have been identified and agreed to take part, local ethics and research governance procedures will be completed. This will include a departmental agreement signed by both the Clinical Director of ED and general paediatrics. The study protocol, departmental agreement and any subsequent modifications of these documents will be reviewed and approved by each site's relevant ethics committees.

Departmental agreement process will be as follows:

For all hospitals

Clinical Directors from ED and general paediatrics will sign a departmental agreement to:

1. Randomisation.
2. Delivery of the intervention to their units.
3. Approach staff members on behalf of the researchers to complete surveys.
4. Extraction of medical record information by the chart auditor.
5. Permission to use this data for the current study and potentially future linked studies, with the understanding that all information will be unidentifiable e.g. using data to compare the results of this study with one undertaken in the future.

Staff members, intervention delivery

ED and general paediatric Clinical Directors will be asked to consult their staff members prior to their decision to participate in the study. They will also be provided with written information about the study.

Patients and their families

This study will not be recruiting patients; however de-identified patient record data will be used to audit clinician practice and to inform quality improvement. Data extraction will be completed by site based trained chart auditors with clinical experience and who have familiarity with clinical records. This person will be a staff member will be authorised to extract data to audit clinical practice in their hospital.

Patient records will be identified retrospectively by the chart auditors. Eligibility will be confirmed and subsequent outcome data will be collected. Only routinely collected information is extracted and the central research team will have access to unidentifiable data only. This maintains patient confidentiality. Finally, the intervention is being delivered at the level of the hospital and not at the level of the patient. There is limited risk of the intervention affecting patients and families' interests adversely.

15.2 Protocol Amendments

This study will be conducted in compliance with the latest approved version of the protocol. Any change to the protocol document or departmental agreement form that affects the scientific intent, study design, or may affect a hospitals willingness to continue participation in the study is considered an amendment, and therefore will be written and filed as an amendment to this protocol and/or departmental agreement form. All such amendments will be submitted to the lead HRECs and/or local ethics committees for approval prior to becoming effective.

Hospital deviations deemed to have a potential impact on the integrity of the study results may result in the hospital being withdrawn from the study. This decision will be the responsibility of the CPI.

16 Protocol publishing

The study protocol will be submitted for publishing in 2016.

17 Reimbursement

All sites will receive a payment of $7000 (Australian Dollars). This is to cover costs of data collection for both intervention and control arms of the study. Other associated costs will be covered by the central site at MCRI e.g. training materials, travel and accommodation to attend training.

18 Declaration of interests

There are no financial conflicts of interest.

19 Dissemination and authorship

The CPIs will have primary responsibility for publication of the results. There are no restrictions on publication. Participant level data will not be made available in publications.

It is expected that site investigators (one from each site) will be acknowledged on the main manuscript. If site investigators fulfil the international requirements for authorship, they will be included as authors in manuscripts.

20 Study management

20.1 Paediatric Research in Emergency Departments International Collaborative (PREDICT)

Founded in 2004, the PREDICT network brings together leading researchers and clinicians in PEM across Australia and New Zealand. It includes all tertiary children's hospital EDs in this region and aims to provide leadership and infrastructure for multicentre research, facilitate collaboration between institutions, health care providers and researchers in PEM. This study will be conducted by the PREDICT network and will involve both sites within the PREDICT network as well as others who are not.

20.2 Study research group

The study will be overseen by a study research group, the membership of which will include the co-principal investigators and at least 4 further co-investigators. The role of this group is to monitor, supervise progress and review at regular intervals. See appendix 2 for study research group membership

20.3 Central study coordination

This will be provided by a team from MCRI, Starship Children's Health and Perth Children's Hospital with co-principal investigators, research site coordinators and administrative support.

20.4 Study management – local

The study will be coordinated at each intervention site by the local champions. Each site (control and intervention) will have a dedicated study research group member who they will work with.

Local champions will be responsible for selecting personnel for data extraction and the dedicated study research group member for the site will be available for assistance, problem solving or advice.

21 Proposed timeline

2016	Jan	Feb	Mar	Apr	May	June	July	Aug	Sept	Oct	Nov	Dec
**All sites**												
Protocol completed												
Contact sites												
Ethics												
Identify clinical lead												
Confirm training day												
Arrange stake-holder meetings												
Ident data collectors												
**2017**	Jan	Feb	Mar	Apr	May	June	July	Aug	Sept	Oct	Nov	Dec
**Intervention sites**												
Training day												
Pre-data collection												
Site visits												
Settling in												
Training of local staff by clinical leads at site												
Regular site contact												
Audit and feedback												
Process evaluation												

2018/19	Dec 2017-March 2018	April-May 2018	Jun-Aug 2018	Sept-Dec 2018	Jan-May 2019	June-Dec 2019	
**Intervention**							
Data collection	Retrospective						
Data (sust)							
**Control**							
Data collection	Retrospective						
Process eval							
Data (sust)							

References

1. Stiell IG, Clement CM, Grimshaw J, Brison RJ, Rowe BH, Schull MJ, et al. Implementation of the Canadian C-Spine Rule: prospective 12 centre cluster randomised trial. *BMJ* 2009;339:b4146.
2. Johnson DW, William Craig W, Brant R, Mitton C, Svenson L, P. KT. A cluster randomized controlled trial comparing three methods of disseminating practice guidelines for children with croup. *Implementation Science* 2006;1:10.
3. Leader S, Kolhase K. Recent trends in severe respiratory syncytial virus (RSV) among US infants, 1997-2000. *Journal of Pediatrics* 2003;143(5s):s127-32.
4. Shay DK. Bronchiolitis-associated hospitalisations among US children, 1980-1996. *JAMA* 1999;282:1440-6.
5.53. Pelletier AJ, Mansbach JM, Camargo CAJ. Direct Medical Costs of Bronchiolitis Hospitalizations in the United States. *Pediatrics* 2006;118:4218-23.
6. Craig E, Anderson P, Jackson C. The Health Status of children and young people in Auckland DHB. Auckland: New Zealand Child and Youth Epidemiology Service, 2008.
7. Roche P, Lambert S, Spencer J. Surveillance of viral pathogens in Australia: Respiratory syncytial virus. Communicable Diseases Intelligence [Internet]. 2003 March 2003; 27(1):[117-22 pp.]. Available from: http://www.health.gov.au/internet/main/publishing.nsf/content/cda-pubs-cdi-2003-cdi2701-htm-cdi2701j.htm.
8. Davison C, Ventre KM, Luchetti M, Randolph AG. Efficacy of interventions for bronchiolitis in critically ill infants: a systematic review and meta-analysis. *Pediatric Critical Care Medicine* 2004;5(5):482-9.
9. Smyth R, Openshaw P. Bronchiolitis. *Lancet* 2006;368:312-22.
10. Babl FE, Sheriff N, Neutze J, Borland M, Oakley E. Bronchiolitis management in pediatric emergency departments in Australia and New Zealand: a PREDICT study. *Pediatric Emergency Care* 2008;24(10):656-8.
11. Oakley E, Borland M, Neutze J, Acworth J, Krieser D, Dalziel S, Davidson A, Donath S, Jachno K, South M, Theophilos T, Babl FE, Paediatric Research in Emergency Departments International Collaborative (PREDICT). Nasogastric hydration versus intravenous hydration for infants with bronchiolitis: a randomised trial. *Lancet Respir Med* 2013; 1: 113–20.
12. Grimshaw J, Eccles MP, Lavis JN, Hill SJ, Squires JE. Knowledge translation of research findings. Implementation Science 2012; 7:50
13. Grimshaw JM, Shirran L, Thomas R, Mowatt G, Fraser C, Bero L, et al. Changing provider behavior: an overview of systematic reviews of interventions. *Med Care* 2001;39(8(S2)):2-45.
14. Bero LA, Grilli R, Grimshaw JM, Harvey E, Oxman AD, Thomson MA. Closing the gap between research and practice: an overview of systematic reviews of interventions to promote the implementation of research findings. *BMJ* 1998;317(7156):465-8.
15. Wohl ME, Chernick V. State of the art: bronchiolitis. *Am Rev Respir Dis* 1978; 118: 759-781.
16. Martinez FD. Respiratory syncytial virus bronchiolitis and the pathogenesis of childhood asthma. *Pediatr Infect Dis J* 2003; 22 (2 Suppl): S76-S82.
17. Field M, Lohr K, eds: Clinical Practice Guidelines: Directions of a New Program. Insitute of Medicine: Washington, DC; 1990: 1 – 160.
18. Edwards SJ, Braunholtz DA, Lilford RJ, Stevens AJ: Ethical issues in the design and conduct of cluster randomised controlled trials. *BMJ* 1999, 318: 1407-1409.
19. Walker AE, Campbell MK, Grimshaw JM: A recruitment strategy for cluster randomized trials in secondary care settings. *J Eval Clin Pract* 2000, 6: 185-192.

Appendix 1

Bronchiolitis guideline PICOT questions:

**1. In infants presenting to hospital what factors in history and physical examination contribute to a differential diagnosis of bronchiolitis?**
**2. In infants presenting to hospital with bronchiolitis, what are the risk factors for admission or severe disease (eg. prolonged hospital stay, intensive care unit admission, death)?**
**3. In infants presenting to hospital or hospitalised with bronchiolitis, does performing a chest X-ray beneficially change medical management or clinically relevant end-points?**
**4. In infants presenting to hospital or hospitalised with bronchiolitis, does performing laboratory tests (blood and/or urine) beneficially change medical management or clinically relevant end-points?**
**5. In infants presenting to hospital or hospitalised with bronchiolitis, does performing virological investigations beneficially change medical management or clinically relevant end-points?**
**6. For infants presenting to hospital or hospitalised with bronchiolitis, does use of a bronchiolitis scoring system beneficially change medical management or clinically relevant end-points?**
**7. For infants presenting to hospital or hospitalised with bronchiolitis, what criteria should be used for safe discharge?**
**8. In infants presenting to hospital or hospitalised with bronchiolitis, does administration of Beta2 Agonists (nebulisation, aerosol, oral or IV) improve clinically relevant end-points?**
**9. In infants presenting to hospital or hospitalised with bronchiolitis, with a personal or family history of atopy, does administration of Beta2 Agonists (nebulisation, aerosol, oral or IV) improve clinically relevant end-points?**
**10. In older infants presenting to hospital or hospitalised with bronchiolitis, does administration of Beta2 Agonists (nebulisation, aerosol, oral or IV) improve clinically relevant end-points?**
**11. In older infants presenting to hospital or hospitalised with bronchiolitis, with a second or subsequent episode/s of bronchiolitis or wheeze, does administration of Beta2 Agonists (nebulisation, aerosol, oral or IV) improve clinically relevant end-points?**
**12. In infants presenting to hospital or hospitalised with bronchiolitis, does administration of adrenaline / epinephrine (nebulisation, IM or IV) improve clinically relevant end-points?**

13. In infants presenting to hospital or hospitalised with bronchiolitis, does administration of nebulised hypertonic saline improve clinically relevant end-points?
14. In infants presenting to hospital or hospitalised with bronchiolitis, does administration of systemic or local glucocorticoids (nebulisation, oral, IM or IV) improve clinically relevant end-points?
15. In infants presenting to hospital or hospitalised with bronchiolitis, with a positive response to Beta2 Agonists, does administration of systemic or local glucocorticoids (nebulisation, oral, IM or IV) improve clinically relevant end-points?
16. In infants presenting to hospital or hospitalised with bronchiolitis, does administration of the combination of systemic or local glucocorticoids (nebulisation, oral, IM or IV) and adrenaline improve clinically relevant end-points?
17. In infants presenting to hospital or hospitalised with bronchiolitis, does administration of supplemental oxygen improve clinically relevant end-points?
18. In infants presenting to hospital or hospitalised with bronchiolitis, what level of oxygen saturation should lead to commencement or discontinuation of supplemental oxygen to improve clinically relevant end-points?
19. In infants hospitalised with bronchiolitis does continuous monitoring of pulse oximetry beneficially change medical management or clinically relevant end-points?
20. In infants hospitalised with bronchiolitis does the use of heated humidified high flow oxygen, or air, via nasal cannula improve clinically relevant end-points?
21. In infants hospitalised with bronchiolitis, does chest physiotherapy improve clinically relevant end-points?
22. In infants hospitalised with bronchiolitis, does suctioning of the nose or naso pharynx improve clinically relevant end-points?
23. In infants hospitalised with bronchiolitis, does deep suctioning in comparison to superficial suctioning beneficially improve clinically relevant end-points?
24. In infants hospitalised with bronchiolitis, does the use of nasal saline drops improve clinically relevant end-points?
25. In infants hospitalised with bronchiolitis, does the use of bubble CPAP improve clinically relevant end-points?
26. In infants hospitalised with bronchiolitis, is provision of home oxygen a safe alternative for management?

**27. In infants presenting to hospital or hospitalised with bronchiolitis, does the use of antibiotic medication improve clinically relevant end-points?**
**28. In infants presenting to hospital or hospitalised with bronchiolitis, does the use azithromycin medication improve clinically relevant end-points?**
**29. In infants presenting to hospital or hospitalised with bronchiolitis, does the use of antibiotic medication in infants who are at risk of developing bronchiectasis, improve clinically relevant end-points?**
**30. In infants presenting to hospital or hospitalised with bronchiolitis, does the use of non-oral hydration improve clinically relevant end-points?**
**31. In infants presenting to hospital or hospitalised with bronchiolitis, what forms of non-oral hydration improve clinically relevant end-points**
**32. In infants presenting to hospital or hospitalised with bronchiolitis, does limiting the volume of non-oral hydration impact on clinical relevant end-points?**
**33. In infants presenting to hospital or hospitalised with bronchiolitis, does infection control practises improve clinically relevant end-points?**

Appendix 2

Contact details of KT bronchiolitis study research group

Name	Role	Contact details
Dr Stuart Dalziel<br>Starship Children's Hospital	Co-Principal Investigator	sdalziel@adhb.govt.nz
A/Prof Ed Oakley<br>Murdoch Children's Research Institute (MCRI) & Royal Children's Hospital (RCH), Melbourne	Co-Principal Investigator	ed.oakley@rch.org.au
Libby Haskell<br>Starship Children's Hospital	Co-Investigator	libbyh@adhb.govt.nz
Dr Emma Tavender<br>MCRI	Co-Investigator	emma.tavender@mcri.edu.au
Sharon O'Brien<br>PREDICT	Co-Investigator	Sharon.Obrien@health.wa.gov.au
Catherine Wilson<br>MCRI	Co-Investigator	Catherine.wilson@mcri.edu.au
