# Supplementary material for: Effectiveness of Targeted Interventions on Treatment of Infants With Bronchiolitis: A Randomized Clinical Trial
Source: JAMA Pediatr. 2021 Apr 12;175(8):797–806. doi: 10.1001/jamapediatrics.2021.0295 (PMC8042564; doi:10.1001/jamapediatrics.2021.0295)
Supplement: Supplement 3. — Statistical Analysis Plan [file jamapediatr-e210295-s003.pdf]

FORM-STAT-04A-01	Statistical Analysis Plan
Version 1.1	

HREC/16/RCHM/84

Knowledge Translation in Australasian Paediatric Acute Care Settings: a multi-centred, cluster-randomised controlled trial comparing a tailored, theory informed Knowledge Translation intervention versus passive dissemination of a bronchiolitis guideline. (PREDICT KT study)

FORM-STAT-04A-01	Statistical Analysis Plan
Version 1.1	

Document Version History

Version Date	Version	Author	Signature	Change Description	Reason/Comment
27-Apr-2018	Draft 1	Francesca Orsini		Initial release.	Not applicable.
10-05-2018	Draft 2	Francesca Orsini		Removed PP analysis<br>Added Fidelity Analysis	Incorporating comments coming from: Stuart, Ed, Libby and the rest of the team.
31-05-2018	Draft 3	Francesca Orsini		Added Cluster Level Analysis as sensitivity analysis.	After CEBU Journal Club realized it was important to compare individual level analysis with cluster level one.
01-06-2018	Draft 4	Francesca Orsini		Subgroup analyses description added.	Incorporating comments coming from: Libby, Emma and Cate at the meeting on 1 June 2018.
10-11-2018	Version 1	Rachel Schembri		Added fidelity analysis and exploratory analysis.	Measure of fidelity finalized and implemented. Exploratory analysis incorporated comments from team.
27-11-2018	Version 1.1	Rachel Schembri		Altered primary analysis	Removed logit link function, as Risk Difference is wanted. GLM with family(binomial) and link(identity) used. Cluster-robust standard errors (vce cluster) used instead of a random effect for the cluster variable: site, as random effect cannot be used with a binomial distribution to get a risk difference.

TABLE OF CONTENTS

LIST OF ABBREVIATIONS 4

1. STUDY OBJECTIVES 5
   1.1. PRIMARY OBJECTIVE 5
   1.2. SECONDARY OBJECTIVES 5
2. BACKGROUND/INTRODUCTION 5
   2.1. STUDY DESIGN 5
   2.2. TREATMENT GROUPS 6
   2.3. STUDY POPULATION 7
   2.4. INTERVENTION 8
   2.5. SAMPLE SIZE 8
   2.6. STUDY PROCEDURE 9
3. POPULATIONS OF ANALYSIS 9
4. OUTCOME VARIABLES 10
   4.1. PRIMARY OUTCOME 10
   4.2. SECONDARY PARAMETERS OUTCOMES 10
   4.3. OTHER PARAMETERS 11
5. STATISTICAL METHODOLOGY 11
   5.1. GENERAL METHODOLOGY 11
   5.2. PRIMARY DATA ANALYSES 14
   5.3. SECONDARY DATA ANALYSES 14
   5.4. EXPLORITORY DATA ANALYSES 16

LIST OF ABBREVIATIONS

AE	Adverse Event
CRF	Case Report Form
CXR	Chest X-Ray
ED	Emergency Department
GCP	Good Clinical Practice
KT	Knowledge Translation
ICU	Intensive Care Unit
ITT	Intent-To-Treat
SAE	Serious Adverse Event
SD	Standard Deviation
SE	Standard Error

1. STUDY OBJECTIVES

1.1. PRIMARY OBJECTIVE

To determine whether a tailored, theory informed Knowledge Translation (KT) intervention is effective in decreasing the use of therapies known to be of no benefit, compared to passive dissemination of a bronchiolitis guideline.

1.2. SECONDARY OBJECTIVES

To evaluate differences in effectiveness of KT intervention strategies versus usual practice:

1. In decreasing length of stay
2. In decreasing cost of hospital stay for infants with bronchiolitis, and in hospitals of differing caseloads (regional vs. metro vs tertiary).

The current Statistical Analysis Plan (SAP) will detail the plan of analysis to answer the primary objective and secondary objective 1.

2. BACKGROUND/INTRODUCTION

For detailed background and rational information refer to Section 4, Study Protocol, Version 1.2 – 2016-07-29.

2.1. STUDY DESIGN

This is a multi-centre, cluster-randomised controlled trial with the hospital being the cluster, including the Emergency Department (ED) and general paediatric staff members involved in the care of infants with bronchiolitis. Clusters have been chosen for the following two reasons: the intervention is targeted to the staff involved in the care of infants with bronchiolitis, and the hospitals represent patient populations in geographical areas, removing the use of an individually randomised design. See Figure 1 for the study process design.

Figure 1 KT bronchiolitis study process design

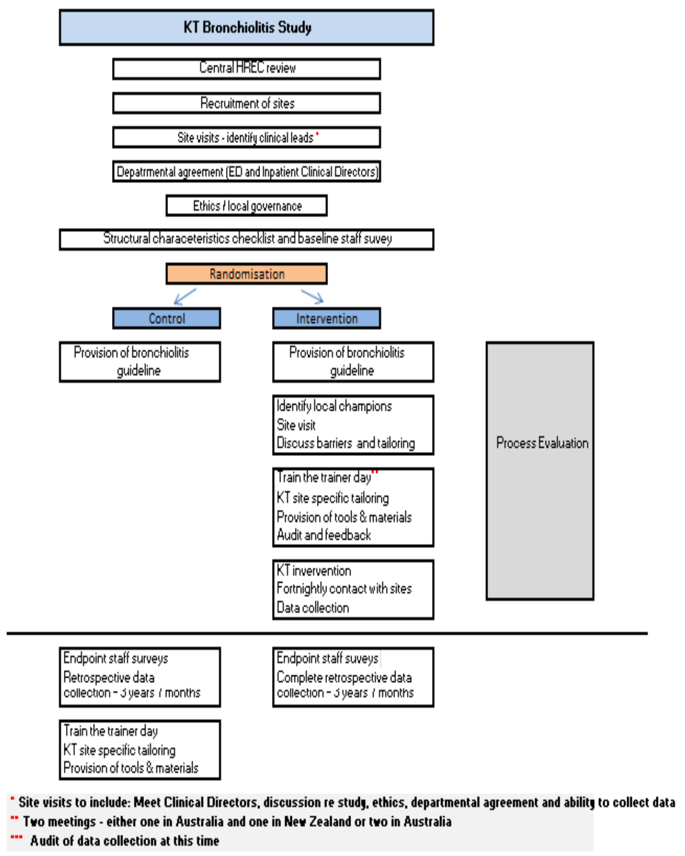

2.2. TREATMENT GROUPS

<u>RANDOMISATION PROCESS</u>

Randomisation of hospitals will be within two stratification factors:

- by country: Australia vs New Zealand
- by paediatric hospital status: tertiary vs secondary hospital

into either KT intervention or control group. Randomisation will be completed using randomisation computer software, created by a statistician who is not affiliated with the study. A potential threat to the validity of this study is if health care personnel from the KT intervention arms and control communicate. The researchers will aim to minimise contamination. The majority of recruited hospitals will be in different regions around Australasia, with significant physical distance between them which will limit some contact between sites.

<u>STUDY GROUPS</u>

Interventions will be classified as tailored, theory informed KT intervention (intervention group) or passive dissemination (control group).

Intervention group

Hospitals randomised to the intervention group will receive:

- Electronic and printed copies of both the full and bedside version of the Australasian Bronchiolitis Guideline.
- Tailored, theory informed intervention built upon by qualitative interviews (from a previous study; HREC 36179A) from a range of hospital types and medical and nursing personnel to determine barriers and enablers in implementing guideline. Intervention components will be tailored to target these identified barriers/enablers.
- A selection of KT interventions that individual hospitals assigned to this group will use. The study's KT implementation team will support clinical leads during the intervention period with regular email and phone contact. It is envisaged that the intervention will include the following core items: training of clinical leads, training of local staff by clinical leads, implementation of promotional material /reminders and audit and feedback. This will occur over a 7 month period with 3 months of this being intensive training.

Control group

Hospitals randomised to the control group will receive:
- Passive dissemination: Electronic and printed copies of both the full and bedside version of the Australasian Bronchiolitis Guideline.

<u>BLINDING</u>

Due to the nature of the intervention, it will not be possible to blind staff members involved in the study to group allocation. This is a potential threat to the validity of the study. As mentioned previously, communication between intervention and control groups will not be actively encouraged but still may occur.

Ideally hospitals will use local data collectors not associated with patient care in ED or the general paediatric inpatient areas and who are not aware of study outcomes. A percentage of data collection at each site will be independently audited to confirm the authenticity of the data. The data collection training will focus on the operational aspects of the study

2.3. STUDY POPULATION

26 hospitals across Australasia (including 6 New Zealand sites) each with an ED census of >135 cases of bronchiolitis per year will be randomly assigned to one of two interventions. Hospitals will be stratified by country (Australia, New Zealand) and the paediatric hospital status (tertiary or secondary providers of paediatric care).

All participating hospitals must fulfil all of the inclusion criteria and none of the exclusion criteria.

Inclusion criteria for sites:

To be eligible for this study, each hospital must:
- Have an ED census of >135 cases of bronchiolitis per year.
- Be willing to participate (either in the control or intervention arm).
- Have a signed departmental agreement from the ED and inpatient clinical directors.
- Have the ability to audit clinical notes (this will be tested during site visits with the principal investigator or designated study personnel).
- Nominate a clinical lead for medical and nursing for the duration of the study.
- Have the ability to recruit medical and nursing staff to complete staff surveys at baseline and endpoint.

Exclusion criteria for sites:
- Inability to audit clinical notes.

- Be averse to participating if randomised to the control arm (control sites will receive the KT training that intervention arm received, at the end of the study).
- Royal Children's Hospital, Melbourne; Princess Margaret, Perth; Starship Children's Health, Auckland. These sites have personnel who have significant lead roles in the study design and implementation or have been integral in the development of the bronchiolitis guideline. Excluding these hospitals will reduce the risk of potential bias in the study results.

Inclusion criteria for nurses are:
- Current ED or general paediatric employee
- On active practice roster
- Registered or enrolled nurse

Inclusion criteria for medical staff are:
- Current ED or general paediatric employee
- On active practice roster
- Registrars, House Officers (or equivalent) or consultants

Exclusion criteria for nursing and medical staff are:
- Students / interns
- Clinicians not currently engaged in clinical practice
- Agency or bank staff (nurses) or locums (medical)

Inclusion criteria of patients:
- Aged less than 12 months (at time of presentation), AND
- A recorded diagnosis of bronchiolitis on discharge from ED to home, OR
- A diagnosis of bronchiolitis on discharge from inpatient area AND a recorded diagnosis of bronchiolitis in ED

There is no exclusion on the basis of co-morbidities or transfer from other health care facilities. However, these two categories will be used for sub-group analyses.

2.4. INTERVENTION

For detailed Intervention components and process evaluation method information refer to Table 1 and Table 2, Study Protocol, Version 1.2 – 2016-07-29.

2.5. SAMPLE SIZE

The primary outcome of this study is correct use of a bronchiolitis guideline in reducing therapies/management known to have no benefit (Chest X-Ray (CXR), salbutamol, adrenaline, antibiotics and glucocorticoids). In the calculation of sample size for this outcome, adjustment has been made for the clustered nature of the design. The aim is to power the study to show an absolute decrease in assignment of intervention/therapies known to be of no benefit of at least 15%. The rationale for selecting this difference is based on justifying the resource intensive intervention. Data from the seven PREDICT sites that participated in the CRIB2 trial (preliminary data on inappropriate interventions: CXR performed, salbutamol given, oral or IV steroids given, antibiotics given, adrenaline given) were used to determine power calculations for this study. This showed that the proportion of individuals who received at least one inappropriate intervention, ranged from 0.36 to 0.61 with a pooled estimate of about 52% [confidence interval based on a random effects model: 0.45 to 0.59].

The sample size calculations took the nature of the outcome (binary variable outcome variable: guideline-conform treatment yes/no) as well as the issue of clustering (heterogeneity in estimated proportions between sites that exceeds variability being explained by random sampling) into account.
The power calculations revealed a necessary group sample size of 1620 individuals, assuming an average cluster size of 135 patients (and 24 sites). These sample size calculations assumed (informed by preliminary data), an intra-cluster correlation coefficient of <0.06 as well as a proportion relative frequency of the primary outcome of 50% in the control arm. The final study sample will include 26 sites to allow for loss to follow-up/loss of participation of one site in each arm of the study.

2.6. STUDY PROCEDURE

Recruitment of hospitals
The co-investigators will be responsible for approaching individual hospitals. Hospitals within the PREDICT network will be approached in the first instance to assess interest, willingness and ability to be involved. Hospitals will be included once a departmental agreement has been signed by both the ED and General Paediatric Clinical Directors (or equivalent). Refer to Inclusion/Exclusion criteria for sites in section above.

Recruitment of medical and nursing staff
Medical and nursing staff (up to 20 clinicians) from ED and general paediatrics areas in the intervention arm and control groups will be invited to complete two questionnaires (at baseline and endpoint). The aim of this is to explore factors that may influence how they manage infants with bronchiolitis. Staff participants will be selected from the active list of medical and nursing staff working in the area. Each staff member will be given a research ID and the central research team will select which IDs are to complete the survey. The site local champion will organise delivery of an invitation letter (which includes approval by the Clinical Director), and the survey to the selected staff members via mail or email. Consent will be implied if a completed survey is returned directly to the central research team. This will be stated clearly in the introduction to the questionnaire. Additional staff will be randomly selected to complete a survey if there are staff who are lost to follow-up. Refer to Inclusion/Exclusion criteria for nursing and medical staff in section above.

Patient data
In order to determine the effect of the intervention on clinical practice outcomes, data extraction from a random selection of patient records will be conducted by chart auditors appointed at each site. Refer to Inclusion/Exclusion criteria for patients in section above.
Retrospective patient data extraction will be undertaken on clinical notes pre and post the release of the bronchiolitis guideline from all enrolled sites. A total of 3 years 7 months of data will be collected (100-135 patients/year/site as detailed below) on relevant outcome measures:

- 1/05/14 – 30/04/2016 - Retrospective chart audit (100 patients/ year) pre KT interventions (2 years)
- 1/5/16 – 30/4/17 – Retrospective chart audit (100 patients) - “washout period” during which bronchiolitis guideline is released (1 year)
- 1/05/17 – 30/11/17 – Retrospective chart audit (150 patients) post KT intervention (7 months)

3. POPULATIONS OF ANALYSIS

The intention-to-treat (ITT) population will be used in the analyses. Patient outcome data will be compared according to the group to which their hospital was randomly allocated, regardless of

compliance to guidelines. This approach preserves the prognostic balance in the study arms achieved by randomisation.

4. OUTCOME VARIABLES

4.1. PRIMARY OUTCOME

Compliance or non-compliance for each patient presentation with the guideline during the first 24 hours following presentation to ED (acute care period), with regards to the use of key therapies/management processes known to have no benefit (CXR, salbutamol, glucocorticoids, antibiotics, epinephrine).

4.2. SECONDARY PARAMETERS OUTCOMES

1. Compliance or non-compliance for each patient presentation with the guideline with regards to the use of key therapies / management processes known to have no benefit (chest x-ray, salbutamol, glucocorticoids, antibiotics, epinephrine):
   a. While in ED
   b. While an inpatient
   c. During total hospitalization

2. Compliance or non-compliance for each patient presentation with guideline recommendations during the first 24 hours following presentation to the ED (acute care period) with regards to the use of:
   a. Chest x-ray
   b. Salbutamol
   c. Glucocorticoids
   d. Antibiotics
   e. Epinephrine

3. Compliance or non-compliance for each patient presentation with guideline recommendations during their total hospitalization with regards to use of:
   a. Chest x-ray
   b. Salbutamol
   c. Glucocorticoids
   d. Antibiotics
   e. Epinephrine

4. Process evaluation including measure of receipt, delivery and acceptability (only for intervention sites - *Fidelity analysis*, section 5.1). In particular, fidelity will be assessed basing on the following information:
   a. Number of presentations given, duration, number of staff attending, information sheets provided
   b. Information on audit and feedback
   c. Promotional materials used e.g. emails, posters, screen savers

5. Length of stay, defined as total time (in days) from presentation to ED to discharge from hospital (or ED if not admitted or inpatient ward if admitted)

6. Death and or intensive care admission

7. Median number of medication doses:
    a. In acute care period
    b. During total hospitalisation

4.3. OTHER PARAMETERS

DEMOGRAPHY AND BASELINE

The following patient data will be collected:

- Date of birth
- Sex
- Ethnicity

OTHER

- Date and time of ED presentation
- Date and time of disposition from ED and where to – admitted or discharged home
- Date and time of discharge from inpatient setting
- Date and time of transfer to intensive care unit
- Date and time of transfer to ward from Intensive Care Unit (ICU)
- Past history
- CXR during hospitalisation, time taken, CXR report
- Salbutamol administration during hospitalisation: number and timing of doses, discharged on salbutamol
- Adrenaline administration during hospitalisation: number and timing of doses
- Glucocorticoid administration during hospitalisation: number and timing of doses
- Antibiotics administration during hospitalisation: number and timing of doses
- Supplementary oxygen during hospitalisation
- High flow during hospitalisation

5. STATISTICAL METHODOLOGY

5.1. GENERAL METHODOLOGY

Data analysis for this study will be performed by Ms Francesca Orsini, an experienced biostatistician who works in the Clinical Epidemiology and Biostatistics Unit (CEBU) at the Murdoch Children's Research Institute (MCRI).

The baseline and demographic characteristics of the hospital and the patients, will be presented for each group using the mean, standard deviation (SD), median and IQR for continuous data and proportions for categorical data.

GENERALIZED LINEAR MIXED (GLM) MODELS

Generalized Linear Mixed Models will be adopted to analyse all the outcomes described in Section 5.2. In particular, the three models will be run.

GLM Model A

This GLM Model will include the stratification factors used during randomization, e.g. country (Australia vs. NZ) and paediatric hospital status (tertiary vs secondary hospital) as fixed effect terms. A random effect term will be included for study site.

GLM Model B

This GLM Model will include the same fixed effect terms as Model A, plus:

- Child sex
- Gestational age less than 37 weeks (yes vs no)
- Chronological age at presentation less than 10 weeks (yes vs no)
- Indigenous ethnicity (yes vs no): defined as Maori, Samoan, Tongan, Niuean, Aboriginal, Torres Strait Islander
- Presence of Co-Morbidities at presentation (yes vs no – sub-group 1)
- Referred from another Hospital OR Representation with Bronchiolitis for the same event (yes vs no – sub-group 2).

GLM Model C

Two different GLM Model C will be run. They will include the same fixed effect and random terms as Model B, and will also include the following fixed effect interaction terms included one at a time with each of the subgroup variables:
- Presence of Co-Morbidities at presentation * intervention group
- Referred From Another Hospital Or Representation With Bronchiolitis * intervention group

Through these models we will examine whether there is evidence that the intervention differed for the subgroups using tests of interaction between intervention and patient factors as follows: subgroup-1 Presence of Co-Morbidities (yes vs no), subgroup-2 Transfer From Other Health Care Facilities Or Representation With Bronchiolitis (yes vs no) (see section SUBGROUP ANALYSIS below). Should any of these fixed effect interaction terms reveal evidence that the intervention effect varies between these groups, specific subgroup estimates and confidence intervals will be presented. As we have not powered the trial to consider subgroups, these analyses are considered exploratory.

SENSITIVITY ANALYSES

The following sensitivity analyses will be run on all the outcomes described in Section 5.2.

Sensitivity 1 - Cluster Level Analysis

Since the clusters are the experimental units of this cluster RCT, we will measure the endpoints of interest (primary outcome and secondary outcomes 1-6) for each site and then compare these between groups as a sensitivity analysis. In particular, the overall observed proportion (of compliance to guidelines) in each group will be obtained by dividing the number of patients for which guidelines were followed (summed across all sites) by the total number of patients in these sites. This proportion is a weighted average of the cluster proportions, with the weights provided by the sample size for each cluster (If these cluster summaries are positively skewed we may consider applying a logarithmic transformation to the proportions prior to analysis. Then, we will take the mean of the log-proportions over clusters, to obtain the log of the geometric mean of the proportion in each treatment arm). We will then carry out a t-test on the observed cluster-level proportions to test null hypothesis of no difference between the groups. The 95% confidence intervals for the risk difference will be calculated.

Sensitivity 2 - Exclusion of sites unable to collect data on patients with length of stay < 3 hours
This sensitivity analysis will exclude those sites where data was unable to be collected on patients with a hospital length of stay less than 3 hours, due to coding not occurring on these patients.

Sensitivity 3 - Handling of Missing Data
Should the percentage of missing data be higher than 10% a multiple imputation analysis will be run, the frequency and patterns of missing data will be examined and sensitivity analyses will be performed to compare the results of analyses restricted to patients with complete data and analyses where those with missing data are considered using multiple imputation techniques. Multiple imputation models will be specified including all variables included in the analysis models and 50 completed data sets will be imputed by chained equations including all the mothers initially randomised.

SUBGROUP ANALYSIS

Sub-Group analysis 1 – Presence of Co-Morbidities
This analysis will examine whether the intervention has differential effects for patients who have co-morbidities versus patients who do not. Presence of Co-Morbidities at presentation (yes vs no) is defined as failure to thrive, OR chronic lung disease, OR congenital heart disease, OR chronic neurological conditions (as collected on question 2.1 of CRF form "Does the infant have any documented past medical history").

Sub-Group analysis 2 – -Referred from Another Hospital or Representation with Bronchiolitis
This analysis will examine whether the intervention has differential effects for patients who were referred from hospital or represented with bronchiolitis versus patients who did not.

	Question	Answer
Patients referred from another hospital are identified as follow:	3.3 "Was the infant referred to hospital?"	- Yes
	3.4 "If referred, where did the infant get referred from?	- Another hospital,
Representation with Bronchiolitis	3.3 "Was the infant referred to hospital?"	- No<br>OR<br>- Unknown
	3.5 If the infant was not referred to hospital, but had been seen prior to presentation by a Doctor for this bronchiolitis episode, where or by whom were they seen?	- Another hospital<br>OR<br>- This hospital

FIDELITY ANALYSIS (ONLY FOR INTERVENTION SITES)
This analysis will examine how fidelity is related to effectiveness. Only Intervention hospitals will be included in this analysis. Process evaluation measures (described in section 4.2, point 4) will be used to assess fidelity (whether the intervention was successfully and consistently delivered as

planned and to whom it was delivered), the quality of implementation, and to characterise contextual factors associated with variation in outcomes.

Fidelity will be scored for each site based on the number of clinical leads, clinical lead attendance at the train-the-trainer day, stakeholder meeting participation, delivery of intervention materials at sites, use of additional materials to deliver intervention, and completion of monthly audits and feedback. Each of these six components is scaled to contribute 4 points to a total fidelity score out of 24.

Fidelity will be analyzed as a continuous measure, for each of the 13 active intervention sites. The relationship between fidelity and the primary outcome (compliance) will be analyzed with a linear regression. Proportion compliant (per site) will be the outcome, and fidelity the predictor. A scatterplot of the data will be produced to observe the relationship, and regression beta coefficient (and 95% confidence interval) reported. Linear regressions will be performed separately for total fidelity score and each of the six factors which make up fidelity.

5.2. PRIMARY DATA ANALYSES

Compliance with the guideline during acute care period (first 24 hours following presentation to ED), with regards to the use of key therapies / management processes known to have no benefit (chest x-ray, salbutamol, glucocorticoids, antibiotics, epinephrine)

Absolute and relative frequencies of patients meeting compliance as per definition of the primary outcome will be calculated and presented by group, overall as well as by department responsible for care at the time of medication (ED, Paediatric, ICU, Other, Unknown, Multiple).
A Generalized Linear Model will be used to estimate the marginal difference in the proportion of bronchiolitis patients treated in accordance with the existing guideline between the study groups. The GLM approach will use the binomial family and employ an identity link function and will calculate a cluster-robust standard error to account for study site. Based on the GLM, risk differences with 95% confidence internals will be computed. GLM models A, B, and C will be run. Cluster level analysis will be run as sensitivity analysis.

5.3. SECONDARY DATA ANALYSES

BINARY SECONDARY OUTCOMES

1a- *Compliance with the guideline while in ED, with regards to use of any therapies*
1b- *Compliance with the guideline while an inpatient, with regards to use of any therapies*
1c- *Compliance with the guideline during total hospitalization, with regards to use of any therapies*
2a- *Compliance with the guideline during acute care period, with regards to the use of CXR*
2b- *Compliance with the guideline during acute care period, with regards to the use of Salbutamol*
2c- *Compliance with the guideline during acute care period, with regards to the use of Glucocorticoids*
2d- *Compliance with the guideline during acute care period, with regards to the use of Antibiotics*
2e- *Compliance with the guideline during acute care period, with regards to the use of Epinephrine*
3a- *Compliance with the guideline during their total hospitalization, with regards to the use of CXR*
3b- *Compliance with the guideline during their total hospitalization, with regards to the use of Salbutamol*
3c- *Compliance with the guideline during their total hospitalization, with regards to the use of Glucocorticoids*
3d- *Compliance with the guideline during their total hospitalization, with regards to the use of Antibiotics*

3e- *Compliance with the guideline during their total hospitalization, with regards to the use of Epinephrine*
6-*Death*
6-*Intensive care admission*
6-*Death and intensive care admission.*

Absolute and relative frequencies of patients meeting compliance as per definition of outcomes 1a-1e, 2a-2e, 3a-3e, and 6 will be calculated and presented by group, overall as well as by department responsible for care at the time of medication (ED, Paediatric, ICU, Other, Unknown, Multiple).
A Generalized Linear Model will be used to estimate the marginal difference in the proportion of bronchiolitis patients treated in accordance with the existing guideline between the study groups. The GLM approach will use the binomial family and employ an identity link function and will calculate a cluster-robust standard error to account for study site. Based on the GLMM, risk differences with 95% confidence internals will be computed. GLM models A, B, and C will be run. Cluster level analysis will be run as sensitivity analysis.

CONTINUOUS SECONDARY OUTCOMES
5- Length of stay
7a- Number of medication doses in acute care period
7b- Number of medication doses in during total hospitalisation

---

If outcomes 5, 7a and 7b are normally distributed, mean and SD will be calculated and presented by group.
A Generalized Linear Mixed Model will be used to estimate the marginal difference in the means between the two groups. The GLMM approach will employ an identity link function and will include random effect terms for study site. Based on the GLMM, differences in means with 95% confidence internals will be computed. GLM models A, B, and C will be run.
If outcomes 5, 7a and 7b are not normally distributed, median, interquartile range (IQR) and range will be calculated and presented by group. Instead of an identity link function, the GLMM approach will employ a log link function (equivalent to a mixed-effects Poisson regression).

Table 1 Summary of outcomes analysis

Outcomes	GLM	Adjustment for covariates	Sensitivity analysis
Binary Outcomes:<br>**primary outcome,** outcome 1a, outcome 1b, outcome 1c, outcome 2a, outcome 2b, outcome 2c, outcome 2d, outcome 2e, outcome 3a, outcome 3b, outcome 3c, outcome 3d, outcome 3e,outcome 6	Comparisons between treatment arms will be made using GLM models, with presentation of risk differences and 95% confidence intervals.<br>Identity Link f.	Model A<br>Model B<br>Model C	Sensitivity 1<br>Sensitivity 2<br>Sensitivity 3 (if more than 10% missing)
Continuous Outcomes:<br>outcome 5, outcome 7a, outcome 7b	Comparisons between treatment arms will be made using GLM models, with presentation of mean differences and 95% confidence intervals<br>Identity link f. (or log link function if not normally distributed)	Model A<br>Model B<br>Model C	Sensitivity 1<br>Sensitivity 2<br>Sensitivity 3 (if more than 10% missing)

5.4. EXPLORITORY DATA ANALYSES

The primary outcome measure of compliance will be investigated for change over time, for the 4 years of study data. A logistic regression will be conducted with compliance as the outcome, and year, randomization group, and a year by group interaction entered into the model.

This data will also be presented graphically, plotting percentage compliance for each year. Year 4 data (post-intervention) will be presented separately for each group, or collapsed into a single group if no between-group differences are found.
