# Supplementary material for: Effectiveness of Targeted Interventions on Treatment of Infants With Bronchiolitis: A Randomized Clinical Trial
Source: JAMA Pediatr. 2021 Apr 12;175(8):797–806. doi: 10.1001/jamapediatrics.2021.0295 (PMC8042564; doi:10.1001/jamapediatrics.2021.0295)
Supplement: Supplement 4. — Data Sharing Statement [file jamapediatr-e210295-s004.pdf]

# Data Sharing Statement

Haskell. Effectiveness of Targeted Interventions on Treatment of Infants With Bronchiolitis. *JAMA Pediatr*. Published April 12, 2021. doi:10.1001/jamapediatrics.2021.0295

**Data**
**Data available:** Yes
**Data types:** Deidentified participant data
**How to access data:** S.Dalziel@auckland.ac.nz
**When available:** With publication

**Supporting Documents**
**Document types:** None

**Additional Information**
**Who can access the data:** Researchers whose proposed use of the data has been approved.
**Types of analyses:** Meta-analysis.
**Mechanisms of data availability:** With investigator support, after approval of a proposal, following ethics approval and with a signed data access agreement.
**Any additional restrictions:** The study used identifiable individual patient data that are subject to restriction, including ethics, consent, and privacy issues. Anonymized data will be available on request with the corresponding author, where possible within these constraints for use. All proposals requesting data access will need to specify how the data will be used and all proposals will need the approval of the trial co-investigator team before data release.
